# Effects of Human Milk Oligosaccharides on the Adult Gut Microbiota and Barrier Function

**DOI:** 10.3390/nu12092808

**Published:** 2020-09-13

**Authors:** Tanja Šuligoj, Louise Kristine Vigsnæs, Pieter Van den Abbeele, Athanasia Apostolou, Katia Karalis, George M. Savva, Bruce McConnell, Nathalie Juge

**Affiliations:** 1 Quadram Institute Bioscience, Gut Microbes and Health Institute Strategic Programme, Norwich Research Park, Norwich NR4 7UQ, UK; tanja.suligoj@quadram.ac.uk; 2 Glycom A/S, Kogle Allé 4, DK-2970 Hørsholm, Denmark; louise.vigsnaes@glycom.com (L.K.V.); bruce.mcconnell@glycom.com (B.M.); 3 Prodigest Bv, B-9052 Gent, Belgium; pieter.vandenabbeele@prodigest.eu; 4 Emulate Inc., 27 Drydock Ave, Boston, MA 02210, USA; nasia.apostolou@emulatebio.com (A.A.); katia.karalis@emulatebio.com (K.K.); 5 Graduate Program, Department of Medicine, School of Health Sciences, National and Kapodistrian University of Athens, 11527 Athens, Greece; 6 Quadram Institute Bioscience, Core Sciences Resources, Norwich Research Park, Norwich NR5 7UQ, UK; george.savva@quadram.ac.uk

**Keywords:** human milk oligosaccharides, adult gut microbiota, gut barrier function, gut-on-chips, SHIME^®^

## Abstract

Human milk oligosaccharides (HMOs) shape the gut microbiota in infants by selectively stimulating the growth of bifidobacteria. Here, we investigated the impact of HMOs on adult gut microbiota and gut barrier function using the Simulator of the Human Intestinal Microbial Ecosystem (SHIME^®^), Caco2 cell lines, and human intestinal gut organoid-on-chips. We showed that fermentation of 2’-O-fucosyllactose (2’FL), lacto-N-neotetraose (LNnT), and combinations thereof (MIX) led to an increase of bifidobacteria, accompanied by an increase of short chain fatty acid (SCFA), in particular butyrate with 2’FL. A significant reduction in paracellular permeability of FITC-dextran probe was observed using Caco2 cell monolayers with fermented 2’FL and MIX, which was accompanied by an increase in claudin-8 gene expression as shown by qPCR, and a reduction in IL-6 as determined by multiplex ELISA. Using gut-on-chips generated from human organoids derived from proximal, transverse, and distal colon biopsies (Colon Intestine-Chips), we showed that claudin-5 was significantly upregulated across all three gut-on-chips following treatment with fermented 2’FL under microfluidic conditions. Taken together, these data show that, in addition to their bifidogenic activity, HMOs have the capacity to modulate immune function and the gut barrier, supporting the potential of HMOs to provide health benefits in adults.

## 1. Introduction

Human milk oligosaccharides (HMOs) are a family of highly diverse structures of unconjugated glycans encompassing over 150 stereospecific oligosaccharide structures present at high concentrations (5–25 g/L) in breast milk. HMOs are composed of a linear or branched backbone containing galactose (Gal), *N*-acetylglucosamine (GlcNAc), and glucose (Glc), which can be decorated with fucose (Fuc) or sialic acid (Sia) residues, depending on the mother’s secretory status [1,2] (Figure S1). The molecular structures and ratios vary among individuals and are dependent on the expression of α1-2-fucosyltransferase (*FUT2*) and α1-3,4-fucosyltransferase (*FUT3*) genes [3] which determine the secretor (Se) and Lewis (Le) status [4]. The *FUT2* gene catalyzes the transfer of Fuc to terminal Gal through α1,2 linkages, whereas the *FUT3* gene transfers Fuc to GlcNAc in type 1 chains through α1–4 linkages. As a result, 2’FL is the most abundant HMO in the milk of secretor women, however, it is not present in the milk of non-secretor women [5,6], whereas Le-negative women lack lacto-N-fucopentaose II (LNFP II) [7]. In infants, only 1–2% of HMOs are absorbed, and the majority of ingested HMOs reach the large intestine where they provide selective substrates for gut bacteria, modulate the immune system, and prevent the epithelial adhesion of intestinal pathogens [8,9,10].

Gut microbiota has a major impact on human health and physiology including the establishment of the mucosal barrier and maintenance of gut homeostasis [11]. HMOs are best known for their prebiotic effects in breast-fed infants, where they exert a strong bifidogenic effect, characterized by the proliferation of *Bifidobacterium infantis*, *B. breve*, or *B. bifidum* strains [12]. HMO metabolism into short chain fatty acids (SCFAs) in infants has drawn a lot of attention in recent years [13,14,15]. HMOs, through cross feeding with butyrate-producing bacteria, induce butyrate production in the colon [16], an essential SCFA metabolite in the human colon. Butyrate is the preferred energy source for colon epithelial cells, contributes to the maintenance of the gut barrier, and displays immunomodulatory properties. During life, the abundance of bifidobacteria decreases from up to 90% of the total colon microbiota in vaginally-delivered breast-fed infants to <5% in the colon of adults, and decreases even more in that of elderly [16]. Furthermore, low bifidobacteria abundance has been linked to GI disorders such as irritable bowel syndrome (IBS), a common GI disorder characterized by abdominal pain or discomfort and altered bowel function which afflicts ~11% of the world population, as well as in patients with antibiotic-associated diarrhea, inflammatory bowel disease (IBD), obesity, and allergies [16,17]. These studies support a role for HMO-induced microbiota-targeted strategies to restore gut homeostasis in adults. A clinical trial showed that 2’-O-fucosyllactose (2’FL) and lacto-N-neotetraose (LNnT) are safe and well tolerated in adults and are modulators of the adult gut microbiota with an increase in bifidobacteria. This suggests that HMO supplementation may be a valuable strategy to modulate human gut microbiota and specifically promotes growth of beneficial bifidobacteria in adults [18]. However, the impact of HMOs on gut barrier function has been largely underexplored.

The intestinal barrier is comprised of a mucus layer covering a single layer of intestinal epithelial cells, which separates bacteria from the underlying submucosa and, as such, is a critical component of gut homeostasis. The intercellular junctional complexes regulate the entry of luminal nutrients, ions, and water while restricting bacteria entry, and thus regulate the barrier function of the epithelium. Microbes can alter epithelial permeability indirectly through effects on host immune cells and the release of cytokines, which can both reduce or enhance barrier function [19]. In the colon, the intestinal mucus barrier forms a bilayer; a loose outer layer that provides an environment for the commensal microbiota and an inner layer that is devoid of them and protects the underlying mucosa from the luminal content [20]. The intestinal mucus layers are built around large highly-glycosylated gel-forming mucin MUC-2, secreted by goblet cells which provide binding sites and nutrients for the bacteria which have adapted to the mucosal environment [21,22]. The gut microbes can affect the intestinal barrier through the regulation of the expression or distribution of tight junction proteins [23] and also through the regulation of mucus secretion and mucin glycosylation [24,25]. An impaired gut barrier is a critical factor in predisposition to intestinal diseases such as IBS [26] and is also associated with diseases in other parts of the body. Therefore, it is critical to develop strategies aimed at restoring or strengthening barrier function in conditions associated with the development of a ”leaky gut” in humans such as ageing, IBS, or IBD. Because mucin *O*-glycans are structurally similar to HMOs, we hypothesized that HMOs can modulate gut barrier function in adults via the gut microbiota.

First, we used the Simulator of the Human Intestinal Microbial Ecosystem (SHIME^®^) in vitro gastrointestinal model to investigate the influence of HMOs on adult gut microbiota composition and function. Using Caco2 cells and advanced gut-on-chip models based on gut organoids derived from human colonic biopsy (Colon Intestine-Chips), then, we showed that HMO-derived bacterial metabolites affect intestinal barrier function. This fundamental knowledge provides scientific-based evidence to aid in the development of strategies to reinforce and strengthen gut barrier function in adults.

## 2. Materials and Methods

All chemicals were obtained from Sigma (St Louis, MO, USA) unless otherwise stated. The HMOs, 2′FL, and LNnT were supplied by Glycom A/S (Hørsholm, Denmark) as white, free-flowing powders of synthetic origin at 94.7% (2′FL) and 97.7% (LNnT) purity, respectively.

### 2.1. Simulator of the Human Intestinal Microbial Ecosystem (SHIME^®^)

The SHIME^®^ in vitro gastrointestinal model (ProDigest-Ghent University, Ghent, Belgium) was used for long-term dynamic fermentation, as previously described [27] (Figure S2). Briefly, the system consisted of three reactors per study arm. The first reactor was of the fill-and-draw principle and simulated food intake and upper gastrointestinal digestion. Peristaltic pumps added nutritional medium (140 mL, 3× per day) and pancreatic/bile juice (60 mL, 3× per day) and subsequently, over time, simulated the stomach and small intestine. The nutritional medium for the SHIME^®^ consisted of (in g/L) arabinogalactan (1.0), arabinoxylan (2.0) (BioActor, Maastricht, The Netherlands), starch (2) (Anco, Roeselare, Belgium), xylan (1.0), pectin (2.0), D-(+)-Glucose (0.4), yeast extract (3.0), peptone (1.0), cysteine (0.5), and commercial pig gastric mucin (4.0). The pancreatic/bile juice contained (in g/L) NaHCO_3_ (12.5), bile salts (6.0) (Difco, Bierbeek, Belgium), and pancreatin (0.9). The remaining 2 reactors simulated the conditions of the proximal and distal colon. These two reactors were constantly stirred, retained a fixed volume (PC = 500 mL and DC = 800 mL), and their pH was constant (PC = 5.6–5.9 and DC = 6.6–6.9). Mucin-covered microcosms were added to all colonic vessels, enabling maintenance of a luminal microbiota and also a specific mucosal microbiota in the colonic regions [28]. Furthermore, fecal samples from a 26-year-old healthy female were prepared within 1 h, according to standard procedures [29], and inoculated at 5 vol% in the colon regions. Fecal samples were collected according to the ethical approval of the University Hospital Ghent (reference number B670201836585). The investigations were carried out following the rules of the Declaration of Helsinki. The study participant gave informed consent. Each anaerobic compartment was continuously stirred at 37 °C and flushed with N2 to ensure anaerobic conditions.

The system ran in the following four stages: (i) The first stage was stabilization. After inoculation of the reactors with a fresh fecal sample, a two-week stabilization period ran which allowed the microbial community to differentiate in the different reactors depending on the local environmental conditions. (ii) The second stage was the control period (C). During this two-week period (w1 and w2), a standard nutrient matrix was dosed into the model for a period of 14 days. The baseline bacterial metabolite activity in the different reactors was determined and used as the control. (iii) The third stage was the treatment period (T). The SHIME^®^ system was operated under normal conditions for 3 weeks (w1, w2, and w3), but with the standard nutrient matrix supplemented with HMOs. The HMOs tested were 2’FL, 2’FL + LNnT (4:1 mass ratio, MIX), and LNnT in a concentration that was equivalent to 10 g per day based on the safety and tolerance clinical trial in healthy adults [18]. The 4:1 mass ratio of the MIX was selected based on the concentrations of 2’FL and LNnT found in human breast milk of secretor mothers [6,30]. Each fermentation was performed once. (iv) The fourth stage was the washout period (W). During the following two-week period (w1 and w2), the SHIME^®^ system ran with the standard nutrient matrix only. Samples from both the proximal and distal reactors were collected three times a week for SCFA analysis and once per week for bacterial enumeration. This included collection during the control period at Weeks 1 and 2 (Cw1, Cw2), the treatment period at Weeks 1 to 3 (Tw1, Tw2, Tw3), and the washout period at weeks 1 and 2 (Ww1, Ww2) (for experimental design and sample collection please see Figure S2).

### 2.2. Analytical Techniques and Molecular Microbial Analyses

All proximal and distal fermentation samples (collected three times a week during SHIME^®^) were analyzed for SCFA concentrations (Figure S2). The concentrations of acetate, propionate, and butyrate in the fermentation samples were measured using gas chromatography, as previously described [31]. The growth of selected bacterial taxa during the SHIME^®^ run was analyzed in samples from the proximal colon collected each week during the control (Cw1 and Cw2), treatment (Tw1&Tw2 and Tw3), and washout (Ww1 and Ww2) periods and bacteria enumerated using qPCR (see Figure S2). Metagenomic DNA was extracted using the CTAB protocol [32]. Quantitative polymerase chain reaction (qPCR) for bifidobacteria and *Blautia coccoides/Eubacterium rectale* [33], as well as Firmicutes and Bacteroidetes [34] were performed, as previously described [33], using specific primers (Table S1).

### 2.3. Fermented Human Milk Oligosaccharide (f-HMO) Sample Processing for Downstream Analysis

Proximal colon fermentation samples were used for testing on Caco2 and gut-on-chip cultures. The basis of the SCFA and gut microbiome analyses, the f-HMO samples were processed as follows: For controls, the 6 samples collected over a two-week control period (Cw1 and Cw2) for each SHIME^®^ run were pooled into one sample, resulting in samples f-2’FL/C, f-LNnT/C, and f-MIX/C. For treatment samples, f-LNnT/T and f-MIX/T, the 9 samples collected over a 3-week treatment period (Tw1, Tw2, and Tw3) were pooled into one sample. For treatment sample f-2’FL/T, the 6 samples collected over the first 2 weeks of treatment were pooled into one sample (f-2’FL/Tw1&2), and the 3 samples collected over the 3rd week of fermentation were pooled into f-2’FL/Tw3. To prepare the f-HMO samples for stimulating the Caco2 cells and gut-on-chips, the f-HMO samples were centrifuged 10 min at 16,200× *g* and filter sterilized (0.22 µm filter).

### 2.4. Treatment of Caco2 Cell Culture on Transwells with f-HMOs

The Caco2 cells, which had been originally isolated from a human colonic tumor, were purchased from Public Health England (ECACC catalogue no. 09042001). The Caco2 cells were cultured in high glucose Dulbecco’s modified Eagle medium supplemented with 10% fetal bovine serum (FBS), 2 mM L-Gln, non-essential amino acids, 100 U/mL penicillin/100 µg/mL streptomycin (Gibco, Waltham, MA, USA), and 20 mM Hepes. The cells (passage number 14) were seeded on polyester transwells with 0.4 µm pore size (Corning, NY, USA). For the 24 h f-HMO stimulation, the Caco2 cells were seeded in 24-well plates with 12 transwell inserts, diameter 6.5 mm, at 0.33 × 10^5^ cells per transwell. For 32 h f-HMO stimulation, the Caco2 cells were seeded on 6-well plates with 6 transwell inserts, diameter 24 mm, at 4.67 × 10^5^ cells per transwell. The cell culture medium was refreshed twice weekly. Following 20 days of Caco2 cell culture on transwells, f-HMOs diluted 1:5 in culture medium were added to the apical side followed by incubation for 24 h. The Na-butyrate control (5 mM) and cell culture medium (BL) were used as controls. For 32 h f-HMO stimulation, the f-HMO containing medium was refreshed after 24 h. The cells were harvested after a total of 24 h or 32 h f-HMO stimulation.

### 2.5. Colon Intestine Chip Culture

#### 2.5.1. Colonic Biopsies

Colonic biopsies were obtained by endoscopy performed at the Quadram Institute, Norfolk and the Norwich University Hospital (NNUH), for diagnostic or clinical management purposes in subjects with polyps. All subjects gave their informed consent for inclusion before they participated in the study. The study was conducted in accordance with the Declaration of Helsinki and the protocol was approved by the Faculty of Medicine and Health Sciences Research Ethics Committee, University of East Anglia (reference number 2016/17 63 HT, approved on 29 March 2017). Biopsies were taken from macroscopically normal regions of proximal, transverse, and distal colon. See supplements for clinical details of the three study participants (Table S2).

#### 2.5.2. Organoid Culture

To generate organoids, colonic crypts were isolated using EDTA chelation, as previously described [35]. Briefly, endoscopy biopsies were first washed with antibiotic and antimycotic solution containing 200 μg/mL primocin (Invivogen, Toulouse, France) in Ca2+ and Mg2+ free PBS. The biopsies were dissected and washed twice with 0.1 mM DTT. Four incubations (5 min each) with ice cold 2 mM EDTA were performed, with shaking to remove debris between the incubations, followed by a final 35 min EDTA incubation at room temperature. Three fractions in ice cold PBS were obtained by vigorous shaking. The crypts were centrifuged at 100× *g* and resuspended in growth factor-reduced Matrigel (Corning Life Sciences, Corning, NY, USA), followed by addition of IntestiCult™ Organoid Growth Medium (Stemcell Technologies, Cambridge, UK), supplemented with 100 μg/mL primocin and 10 μM Y-27632 (RHO/ROCK pathway inhibitor, inhibits ROCK1 and ROCK2, Stemcell Technologies, Cambridge, UK). The medium contained Y-27632 for 2 days after crypt isolation and following organoid passage. The organoid culture was maintained by changing the Intesticult medium every 2 days. Passaging was performed every 7–10 days by incubating the organoids in polymerized Matrigel in Cell Recovery Solution (Corning, NY, USA) for 45 min, at 4 °C, followed by mechanical dissociation, and the culture typically expanded 1:4.

#### 2.5.3. Colon Intestine-Chips

To establish Colon Intestine-Chips, colonic organoids were seeded on S-1 chips (Emulate Inc., Boston, MA, USA) and processed as follows [36](Apostolou et al., unpublished): Chips were washed with 70% ethanol, cell culture water, and ER2 reagent (Emulate Inc.), and then activated with ER1 (Emulate Inc.) working solution (0.5 mg/mL ER1 powder in ER2 solution) using 20 min UV light treatment (Nailstar, NS-01 EU/UK). The chips were, then, washed with ER2 and PBS and the inner surfaces of both channels and the porous PDMS membrane coated with extracellular matrix proteins (ECM). The top channel was coated with 200 µg/mL collagen IV and 100 µg/mL growth factor reduced Matrigel in PBS. The bottom channel was coated with 200 µg/mL collagen IV and 30 µg/mL fibronectin (Corning, NY, USA) in PBS and incubated in a humidified 37 °C incubator overnight. Then, the chips were washed in PBS and with the respective cell culture media for the top and bottom channel. Human intestinal microvascular endothelial cells (HIMECs, Cell Systems, Kirkland, WA, USA) (30 µL/chip) were seeded at a cell density of 8 × 10^6^ cells/mL on the lower side of the ECM-coated porous membrane in endothelial cell growth medium (EGM-MV2, PromoCell, Heidelberg, Germany). The EGM-MV2 medium contained human epidermal growth factor (hEGF 5 ng/mL), hydrocortisone (0.2 µg/mL), vascular endothelial growth factor 165 (VEGF 0.5 ng/mL), human basic fibroblastic growth factor (hBFGF 10 ng/mL), R3-insulin-like growth factor-1 (20 ng/mL), ascorbic acid (1 µg/mL), and 5% heat inactivated FBS. Chips were inverted and incubated for up to 2 h, at 37 °C, to promote HIMEC cell adhesion to the membrane before the epithelial cells were seeded. Colon organoids passage 4 or 5 were isolated from Matrigel in Cell Recovery Solution for 45 min on ice, centrifuged at 250× *g*, at 4 °C, and fragmented in Trypsin/EDTA 0.25% supplemented with 10 μM Y-27632, for 1.5–2 min, in a 37 °C water bath. Then, the digestion mix (V = 0.5 mL for the sample seeded on 24-well plate was diluted with 10 mL advanced DMEM/F12 (Gibco, Waltham, MA, USA) and centrifuged at 250× *g*, at 4 °C. The epithelial cell pellet was resuspended in IntestiCult™ Organoid Growth Medium supplemented with primocin and Y-27632 of which 40 μL was used to seed each chip, infused into the top channel, and incubated overnight at 37 °C to promote cell adhesion. The following day, both channels were washed once with respective media which had been degassed (Intesticult medium for top and EGM-MV2 for bottom channel) to remove non-adherent cells. The chips were attached to Pod™ portable modules (Emulate Inc.) and connected to the automated culture system ZOË™ (Emulate Inc.). The chips were perfused at 60 μL/h with Intesticult medium supplemented with 100 μg/mL primocin in the top channel and with EGM-MV2 medium supplemented with 500 μg/mL primocin in the bottom channel. The Intesticult medium also contained 10 μM Y-27632, but only for the first day following connection of chips to the pods. The vacuum-generated periodic stretch was set at 2% strain, 0.15 Hz on day 3, and then increased to 10% strain, 0.15 Hz on day 4 to simulate peristalsis-like motions. The media were refreshed every 2 days for 7 days until f-HMO treatment.

#### 2.5.4. Apparent Permeability Measurement

The epithelial barrier integrity of Colon Intestine-Chips was assessed, on days 2, 5, and 7 post cell seeding on chips, using the FITC-dextran MW 4 kDa (FD4). Briefly, FD4 was added in the epithelial medium (top channel) at a concentration of 20 μg/mL. The dye diffused into the vascular channel was monitored by measuring the absorbance of the basal channel effluents at 495–525 nm. For the calculation of the apparent permeability (Papp), the following mathematical formula was used:

Papp = (V_rec_ × dC_rec_)/(A × dt × C_don, t = 0_)

where Papp is the apparent permeability (cm/s); V_rec_ the volume of the retrieved effluent from the vascular channel (mL); dC_rec_ the difference in dye concentration between the effluent and the input medium of the vascular channel (mg/mL); A the surface on which the diffusion of the dye occurs (cm^2^); dt the time period during which the Papp is assessed (s); and C_don, t = 0_ the concentration of the dye in the dosing epithelial medium (mg/mL).

### 2.6. Treatment of the Colon Intestine-Chips with Fermented HMOs (f-HMOs)

The Colon Intestine-Chips were stimulated apically with f-HMOs diluted 1:5 in IntestiCult™ Organoid Growth Medium supplemented with 100 μg/mL primocin. Basolateral media was EGM-MV2 medium supplemented with 500 μg/mL primocin. The flow rate was 30 µL/h for the duration of the treatment. The experiment was performed in triplicate for proximal and distal Colon Intestine-Chips and in duplicate for the transverse Colon Intestine-Chips. After 24 h incubation, the media effluents of the top and bottom channels were collected for cytokine analysis. The epithelial and endothelial media were refreshed, and the stimulation continued for an additional 8 h, at 60 µL/h. The content of the apical chamber was collected in 500 µL Trizol per chip and the replicate chips were pooled into one sample for subsequent RNA extraction. This experiment was performed on three gut-on-chips, i.e., one donor each for proximal, transverse, and distal Colon Intestine-Chips.

### 2.7. Permeability Analysis Using FITC-Dextran Probe

For the permeability assays, the Caco2 cells grown on transwells were stimulated with f-HMOs for 24 h, and then the medium containing f-HMO refreshed. FD4 at 10 mg/mL in Ca2+ and Mg2+ free PBS was added on the apical site of the transwells, at a final concertation of 2.3 mg/mL FD4. Following 8 h incubation, the basolateral medium was collected and kept at −80 °C, in the dark, until analysis. For quantification, a standard curve was prepared with FD4 dilutions in culture medium with concentrations ranging from 0 to 5 µg/mL. Three separate experiments were conducted and a minimum of three and up to six replicates for each sample were used depending on the experimental conditions (as shown in Section 3).

### 2.8. Cytokine Analysis

For the Caco2 cell transwell experiments, f-HMOs were incubated for 24 h and the apical and basolateral media were harvested for cytokine analysis. Cytokine secretion was quantified using Meso Scale Discovery (MSD, Rockville, MD, USA). IL-6, IL-8, and GRO-α were quantified simultaneously on one plate using the MSD U-plex Biomarker Group 1 (hu) assay. TGF-β1, -2, and -3 were quantified simultaneously using the MSD U-PLEX TGF-β Combo (hu) assay. The plates were read using the 1300 Meso QickPlex SQ 120 (MSD, Rockville, MD, USA). The data were expressed as total cytokine amounts secreted (in pg).

For the gut-on-chip experiments, apical and basolateral effluents from Colon Intestine-Chips were analyzed for IL-6 and IL-8 secretion using ELISA MAX Standard Set Human IL-6 (Biolegend, San Diego, CA, USA) and Human IL-8 (CXCL8) Standard ABTS ELISA Development Kit (Peprotech, London, UK), according to the manufacturer’s instructions. The IL-6 and IL-8 amounts were expressed as pg.

### 2.9. RNA Extraction and Quantitative RT-PCR

For the Caco2 cell experiments, cells were washed twice in Ca2+ and Mg2+ free PBS, replicates pooled and lysed in Trizol (Invitrogen, Carlsbad, CA, USA). Similarly, for the gut-on-chip experiments, the replicate samples were pooled and lysed in Trizol and RNA extracted from Trizol homogenates, following the manufacturer’s instructions (Invitrogen). DNA digestion was carried out using RNase-free DNase set (Qiagen, Hilden, Germany) and RNA clean-up with a RNeasy MinElute kit (Qiagen, Hilden, Germany). The RNA yield was measured with Nanodrop and, when needed, the RNA quality was assessed with an Agilent 4200 TapeStation system using High Sensitivity D1000 ScreenTape Assay (Agilent), see results in Figure S3. Reverse transcription was performed with a QuantiTect RT kit (Qiagen, Hilden, Germany).

Quantitative RT-PCR (qRT-PCR) was performed using an Applied Biosystems^®^ 7500 Real-Time PCR System (Thermo Fischer Scientific, Waltham, MA, USA). Primers were purchased from Qiagen or commercially synthesized by Eurofins (Luxembourg) (Table S1). The cDNA (12.5 ng or 20 ng) was amplified in 25 μL reaction containing 0.5 μM of each primer and 12.5 μL of 2× QuantiTect SYBR Green PCR Master Mix (Qiagen, Hilden, Germany). The PCR product specificity was confirmed by melt curve analysis. GAPDH, RPS13, and actin were used as housekeeping genes to normalize the data. Gene expression results are shown using 2^(−∆∆Ct) method for each of the reference genes and their average. The 2^(−∆∆Ct) method was used for a relative quantification of qRT-PCR data. Fold changes greater than 2 were taken to indicate relative upregulation of gene when comparing treatment to its control sample, whereas values ≤0.5 indicated downregulation. No effect on gene expression was considered for values between 2.0 and 0.5.

### 2.10. Statistical Analyses

Throughout, statistical analyses were conducted by comparing outcomes across treatments or between samples before and during treatment phases. Statistical tests were only conducted where units of observation were each independently treated with f-HMOs and subsequently independently assayed. The details of specific statistical analyses follow.

For the permeability analysis, FITC-dextran concentration was log-transformed before analysis. Each of three independent experiments was analyzed separately, as well as being pooled into a single analysis. For separate experiments, t-tests were used to compare FITC-dextran across specific treatment groups. For the pooled analysis, a linear mixed model was used to control for the random effect (random intercept) of the experiment on FITC-dextran permeability. The effect of each treatment as compared with basolateral medium was extracted from the model, as well as the difference between f-HMOs sampled from the control phase as compared with the treatment phase. For the cytokine secretion, differences in mean cytokine secretion between groups between treatments were estimated using linear regression models after log-transformation of each outcome.

For the gut-on-chip experiments, *p*-values for differences between control and treatment values of total IL-6 and total IL-8 were calculated using Welch’s *t*-test.

Throughout, all *p*-values were subsequently adjusted with a multiplicity correction (within each experiment) to reflect a false discovery rate (FDR) using the method of Benjamini and Hochberg [37].

All statistical analysis was performed using R statistical software v3.6.0 [38] with the lme4 v1.1.2 [39] and lmerTest v3.1-0 [40] packages.

## 3. Results

### 3.1. Supplementation of 2’FL and LNnT Modulates Bacterial Taxa and Metabolite Profiles in the Adult Gut Microbiota

The impact of HMOs on the metabolite profile and composition of the gut microbiota from a healthy adult donor was determined using the SHIME^®^ model, a multi-compartment dynamic simulator of the human gut [41]. The retention time and pH of the vessels were chosen to resemble in vivo conditions in the proximal and distal part of the colon. Prior to the SHIME^®^ experiment, short-term colonic simulations (48 h) were carried out with fecal samples from five different healthy human adults to assess the potential interindividual differences (Figure S4). This revealed that for each of the five donors tested, the 2FL/LNnT mixture in a 4:1 ratio (*w/w*) similarly decreased pH (due to increased acetate, propionate, and butyrate production), while increasing gas production and exerting bifidogenic effects. Therefore, knowing that the mechanism under investigation was not highly prone to interindividual differences, a random donor could be selected for the long-term SHIME^®^ experiment that was used to assay the impact of 2’FL, LnNT, and MIX HMOs over a three-week treatment period under controlled conditions (for detail on the study design, please see Materials and Methods). Interindividual differences were further reduced in such long-term model, as the factors that drove interindividual differences, i.e., differences in baseline diet or physiological parameters (pH, transit time, enzyme levels, and bile salt levels) were highly standardized.

**Figure 1:**
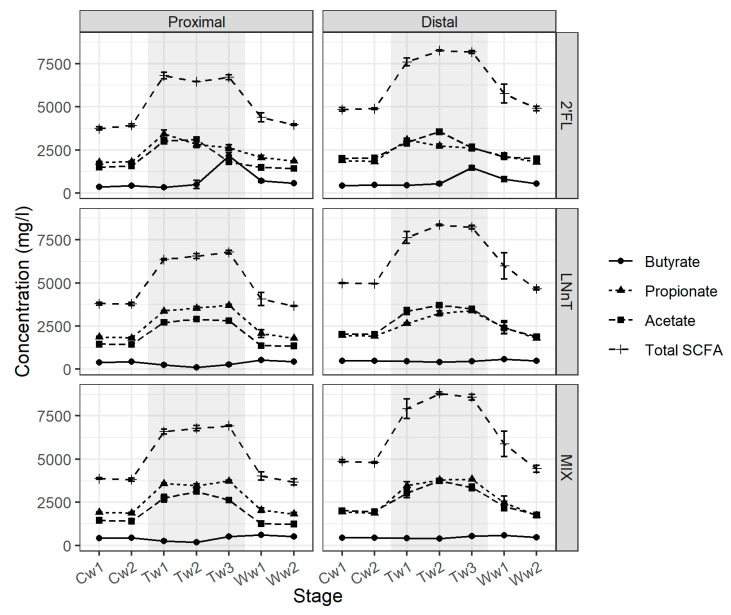
Impact of human milk oligosaccharides (HMOs) on short chain fatty acid (SCFA) production using the Simulator of the Human Intestinal Microbial Ecosystem (SHIME^®^) model. Absolute values of acetic acid, propionic acid, butyric acid, and total SCFA associated with 2’FL (**top panel**), LNnT (**middle panel**), and MIX (**bottom panel**) in the proximal and distal colon reactors are presented. Samples for SCFA analysis were collected during two weeks of control period (Cw1 and Cw2), three weeks of treatment period (Tw1, Tw2, and Tw3), and two weeks of washout period (Ww1 and Ww2). Three samples were collected each week. Error bars correspond to standard errors calculated from the 3 measurements per relevant week.

Treatment with 2’FL immediately increased total SCFA production in the proximal colon, mainly due to a near doubling in concentration of acetate and propionate as compared with the control period (Figure 1).

However, during Week 3, a shift in the metabolite profile was observed with a decrease in the concentration for acetate and propionate to levels close to that of the control period for acetate. This was accompanied by a four-fold increase in the concentration of butyrate as compared with the control period. During the washout period, values for all three SCFAs returned to control levels. A similar SCFA profile was observed for the 2’FL treatment in the distal colon with an increase in the concentration of acetate and propionate during the treatment period, and during Week 3 for butyrate as compared with the control period (Figure 1).

**Figure 2:**
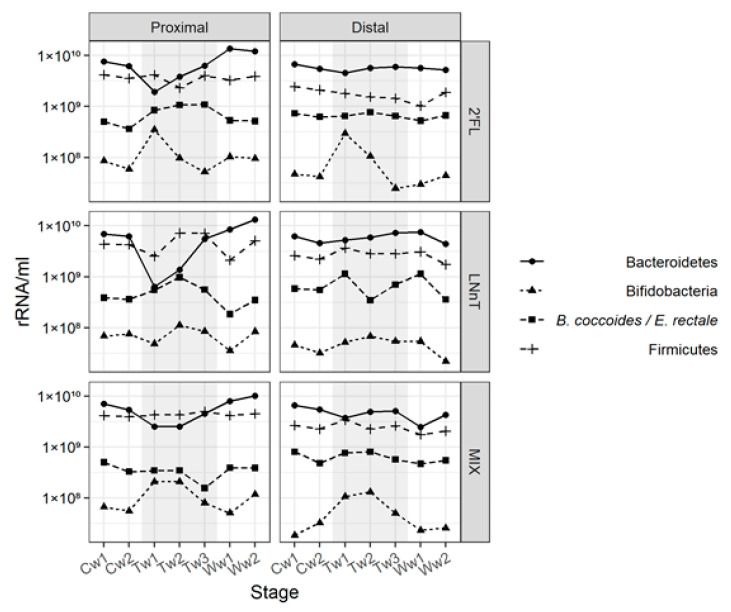
Impact of HMOs, 2’FL (**top panel**), LNnT (**middle panel**), and MIX (**bottom panel**), on microbial populations in the SHIME^®^ model. The relative proportion of Bifidobacterium, *B. coccoides/E. rectale*, Firmicutes and Bacteroidetes in the lumen of the proximal and distal colon vessel was determined by qPCR. Samples for qPCR analysis were collected once a week during two weeks of control period (Cw1 and Cw2), three weeks of treatment period (Tw1, Tw2 and Tw3), and two weeks of washout period (Ww1 and Ww2).

In the proximal vessel, 2’FL had an impact on the *Bifidobacterium* population, increasing the bacterial level as compared with the control period, mainly during the first week of treatment. There was also an increase in *B. coccoides*/*E. rectale* levels as compared with the control period, but the 2’FL treatment did not impact the overall level of Bacteroidetes or Firmicutes. A similar profile was observed for the distal vessel, except for the *B. coccoides/E. rectale* levels, which showed no change as compared with the control (Figure 2).

Treatment with MIX and LNnT also immediately increased total SCFA concentration in the proximal colon region, due to a significant increase in the concentration of acetate and propionate, with a magnitude similar to the effect of the 2’FL treatment. However, in contrast to 2’FL, the increased levels continued throughout the treatment period, and the concentrations of butyrate did not change significantly during treatment as compared with the control. As with 2’FL, the concentrations of acetate and propionate returned to the control levels during washout. A similar metabolite profile was shown for the distal colon (Figure 1).

In the proximal and distal colon vessels, the 2’FL and MIX treatments led to a substantial initial increase of bifidobacteria (>0.5 log copies/mL) after one week of treatment of 0.77/0.85 and 0.58/0.51 log copies/mL in proximal/distal colo, respectively. For 2’FL, there was a decrease in the bifidobacteria levels during the second (−0.56 log copies/mL) and third week (−0.63 log copies/mL) of treatment for the proximal and distal colon, respectively. The 2’FL, LNnT, and MIX treatments caused a decrease in the Bacteroidetes level in the proximal vessel during the first week of the treatment of 0.51, 0.99, and 0.33 log copies/mL, respectively (Figure 2). The decrease of Bacteroidetes in proximal colon appeared to occur at the profit of bifidobacteria for 2’FL and MIX. For 2’FL and LNnT, there was a concomitant increase in *B. coccoides*/*E. rectale* of 0.37 and 0.18 log copies/mL, respectively (Figure 2). It is of note that the Bacteroidetes levels in the proximal colon were restored to that of the control period in the third week of treatment. In the distal vessels, the growth pattern was similar to that observed in the proximal vessels for all HMO tested, except for the Bacteroidetes level which remained relatively constant throughout the treatments (Figure 2).

On the basis of the metabolite and bacterial growth profiles, f-HMOs from the proximal colon were used in the remainder of the study. Fermented LNnT and MIX (f-LNnT and f-MIX) were used after the three-week treatment period (by pooling Weeks 1, 2, and 3). For 2’FL, samples from Week 3 fermentation (f-2’FL/Tw3) were kept separate from Week 1 to Week 2 which were pooled into one sample (f-2’FL/Tw1&2). The samples analyzed in the remainder of the study, therefore, included f-2’FL/C, f-2’FL/Tw1&2, f-2’FL/Tw3, f-LNnT/C, f-LNnT/T, f-MIX/C, and f-MIX/T.

### 3.2. Effect of Fermented HMOs on Epithelial Permeability and Cytokine Production Using Caco2 Cell Monolayers

To assay the impact of f-HMOs on the epithelium barrier, FD4 permeability assays were carried out on differentiated Caco2 cell monolayers after 24 h treatment with f-2’FL/C, f-2’FL/Tw1&2, f-2’FL/Tw3, f-LNnT/C, f-LNnT/T, f-MIX/C, and f-MIX/T. The results from three separate permeability experiments are shown in in Figure S5.

**Figure 3:**
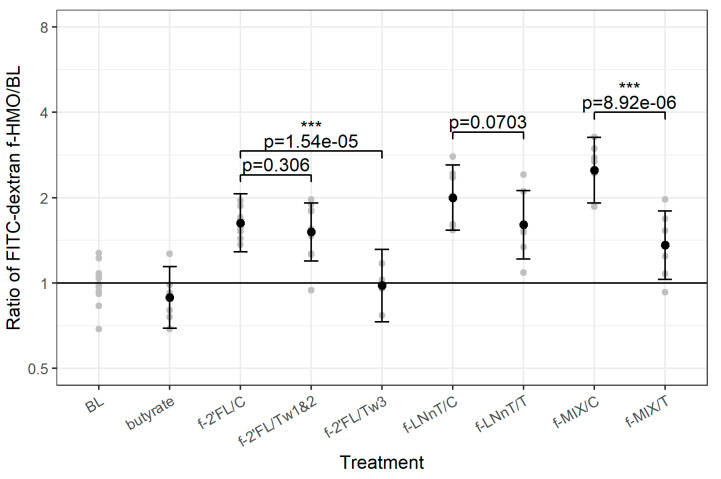
Effect of f-HMO treatment on FD4 permeability on Caco2 cells. The data show results for FD4 concentration in basolateral medium (µg/mL) of f-2’FL/C, f-2’FL/Tw1&2, f-2’FL/Tw3, f-LNnT/C, f-LNnT/T, f-MIX/C, and f-MIX/T, medium-only treated Caco2 cells (BL) and butyrate as the control. Comparison of FD4 permeability for each f-HMO to BL is shown. Grey points represent individual replicates. Black points and error bars (95% confidence levels) represent estimates of treatment effect as compared with BL, with outcomes scaled such that the horizontal line at y = 1 represents the average BL level. p-values correspond to pairwise comparison of FD4 permeability for each of the following f-HMO sample pairs: f-2’FL/C versus f-2’FL/Tw1&2, f-2’FL/C versus f-2’FL/Tw3, f-LNnT/C versus f-LNnT/T, and f-MIX/C versus f-MIX/T. The *p*-values shown for each treatment vs. control comparison are FDR corrected (see text for details). *** shown for *p* < 0.001.

To compare the permeability results across the three experiments, the data were normalized to the Caco2 monolayer treated with medium only (BL). All three bacterial culture supernatants (f-2’FL/C, f-LNnT/C, and f-MIX/C) showed an increased permeability as compared with the Caco2 monolayer treated with the cell culture medium (BL). A similar profile was observed for all treatment samples with the exception of f-2’FL/Tw3 and butyrate control which showed no significant effect on permeability as compared with BL (pooled data across experiments shown in Figure 3, data from individual experiments shown in Figure S5). There were significant differences between f-2’FL/C and f-2’FL/Tw3 (*p* = 1.54 × 10^−5^) and between f-MIX/C and f-MIX/T (*p* < 8.92 × 10^−6^), leading to a significant decrease in permeability and a trend in reduction between f-LNnT/C and f-LNnT/T (*p* = 0.0703), but there was no significant difference between f-2’FL/C and f-2’FL/Tw1&2 (Figure 3). These results indicate a reduction in paracellular transport of FD4 upon treatment with f-2’FL/Tw3 and f-MIX/T as compared with their respective controls (ratios between treatment and control of 0.59 and 0.56, respectively, Table S3). The permeability data obtained for f-2’FL/C as compared with f-2’FL/Tw3 (*p* < 1.54 × 10^−5^) and f-2’FL to Tw1&2 (*p* = 0.306) suggest that the bacterial metabolites produced from longer fermentation period have a more profound effect on the strengthening of the gut barrier (Figure 3).

**Figure 4:**
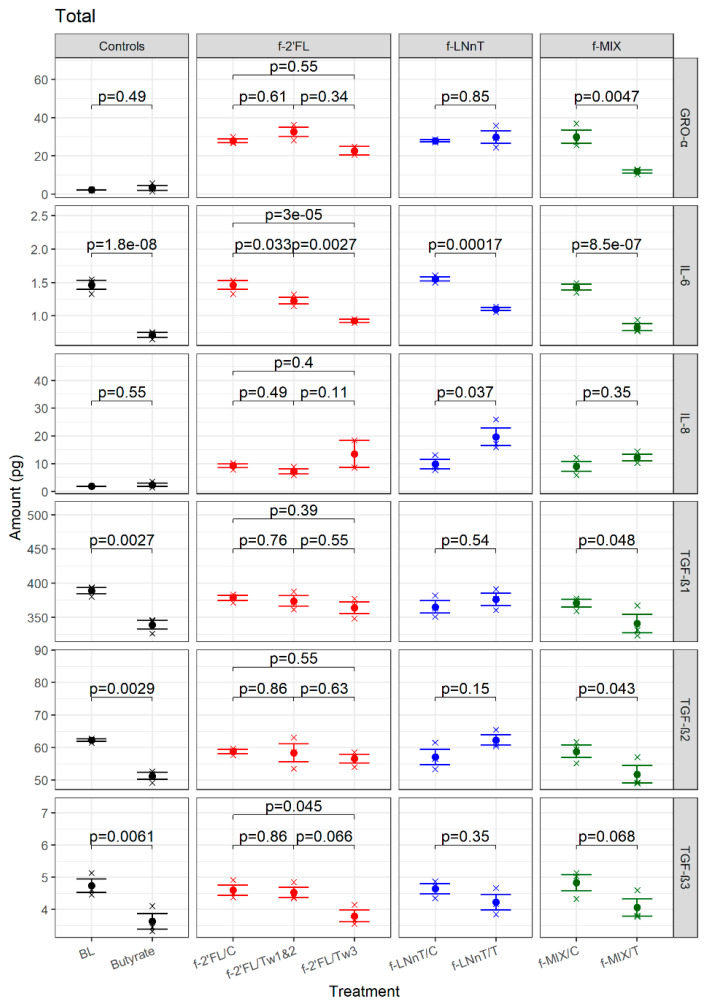
Impact of f-HMO treatment on cytokine secretion of Caco2 cells. Caco2 cells grown on transwells were treated with f-2’FL, f-LNnT, and f-MIX samples, for 24 h. Total amount of secreted cytokines GRO-α, IL-6, IL-8, and TGF-β1–3 are shown (pg). Medium only treated cells (BL) and 5 mM butyrate-treated cells were included as controls. The *p*-values shown alongside each comparison are FDR-corrected (see Section 2 for details).

The impact of f-HMOs on cytokine secretion in the apical and basolateral culture media of Caco2 cells was compared under each treatment using customized multiplex assay of 10 cytokines selected, based on literature [42,43,44,45,46,47]. The MSD multiplex assays are based on multi-array technology which allows simultaneous detection of up to 10 analytes in single immunoassay within a single, small-volume sample (25 µL). Cell culture medium only (BL) and Caco2 cells treated with butyrate (5 mM) were included as the controls. The target cytokines included IL-6, IL-8, GRO-α, TGF-β1, TGF-β2, and TGF-β3. The results are expressed as the total amount of basolateral and apical cytokines secreted (Figure 4).

IL-6 secreted by the CaCo2 cells treated with the controls (f-2’FL/C, f-LNnT/C, and f-MIX/C) or the culture medium (BL) were all in the same range (about 1.5 pg). the butyrate-treated Caco2 cells secreted significantly less IL-6 (0.49-fold) than BL (*p* = 1.79 × 10^−8^, Table S4). A reduction in IL-6 secretion was also observed in the Caco2 cells treated with f-HMOs as compared with their respective controls. This trend was statistically significant for all f-HMO sample pairs under study (Table S4).

The effect of f-HMO treatment on IL-8 secretion by the Caco2 cells was only observed for the f-LNnT sample pair. Fermented LNnT/T led to a significant increase in IL-8 secretion as compared with f-LNnT/C (*p* = 0.0368). Of note, IL-8 secretion of the Caco2 cells treated with control samples (f-2’FL/C, f-LNnT/C, and f-MIX/C) were all in the range of 10 pg as compared with 1.8 pg for BL and 2.4 pg for butyrate-treated cells.

The effect of f-HMO treatment on GRO-α secretion by Caco2 cells was only observed for the f-MIX sample pair. The fermented MIX/T-treated Caco2 cells showed a significant reduction in GRO-α secretion as compared with f-MIX/C-treated cells (*p* = 0.00471). There was no observed effect between the control and treatment f-2’FL and f-LNnT sample pairs or between butyrate-treated cells and BL. Of note, GRO-α secretion of BL and butyrate-treated Caco2 cells was in the range of 2–3 pg, whereas treatment with f-HMO controls (f-2’FL/C, f-LNnT/C, and f-MIX/C) resulted in GRO-α secretion in the range of 25–30 pg.

All f-HMO controls (f-2’FL/C, f-LNnT/C, and f-MIX/C) triggered TGF-β secretion in the same range as the BL-treated Caco2 cells (about 370, 60, and 4.7 pg for TGF-β1, -2, and -3). A significant reduction in secretion of TGF-β1, (*p* = 0.00267), TGF-β2 (*p* = 0.00285), and TGF-β3 (*p* = 0.00612) was observed for butyrate versus BL-treated cells. TGF-β1 and TGF-β2 secretion was also significantly reduced when the Caco2 cells were treated with f-MIX/T as compared with f-MIX/C (*p* = 0.0476 and *p* = 0.0434, respectively), and there was a trend in TGF-β3 reduction (*p* = 0.0676). There was no observed effect of f-2’FL treatments (Weeks 1 and 2, and Week 3) or the f-LNnT treatment on TGFβ-1 and TGFβ-2 secretion. A reduction of TGFβ-3 was observed following treatment with f-2’FL/Tw3 (*p* = 0.0446 for comparison with f-2’FL/C) but no significant effect was observed for f-LNnT.

It is of note that for GRO-α and TGF-β1, -2, and -3, the total cytokine secretion following f-HMOs stimulation reflects the amounts of cytokine secreted basolaterally. This was not the case for IL-6 or IL-8 where the apical cytokine secretion dominated. Individual apical and basolateral amounts for each cytokine are shown in Figures S6 and S7, respectively.

### 3.3. Effect of Fermented HMOs on Gene Expression in Caco2 Cells and Gut-On-Chip Model Systems

On the basis of the results of FD4 permeability following f-HMO treatment on Caco2 monolayers, the effect of f-2’FL and f-MIX was assessed on expression of genes involved in promoting the integrity of barrier function (CLDN-1, -2, -3, -4, -5, -8, ZO-1, and MUC-2) and candidate genes for assessment of immune response (IL-6, IL-8, and GRO-α). Three housekeeping genes, GAPDH, RPS13, and actin were used for data normalization. The Caco2 cells grown on transwells were treated with f-2’FL/Tw3, f-2’FL/C, f-MIX/T, and f-MIX/C for 24 h or 32 h and gene expression analyzed by qRT-PCR.

**Figure 5:**
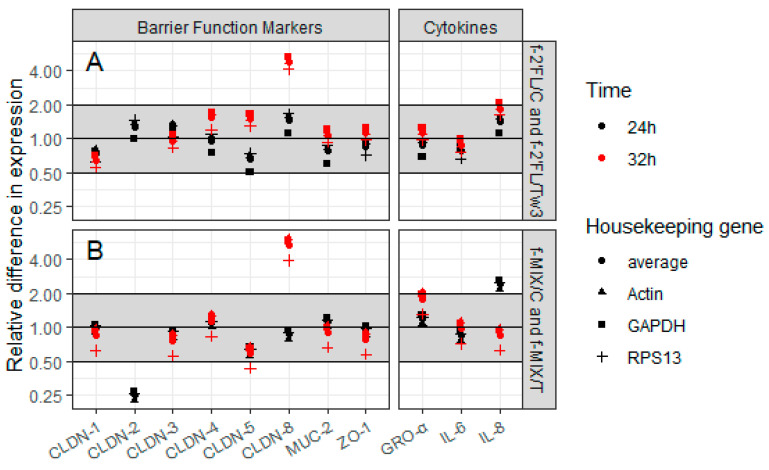
Effect of f-HMO treatment on gene expression of barrier function proteins and gene expression of cytokines of Caco2. The 2^(−∆∆Ct) method data are shown for Caco2 cells grown on transwells that were treated with f-2’FL (**A**) or f-MIX (**B**) for 24 h (black) and 32 h (red). Actin, GAPDH, and RPS13 were used as reference genes to normalize the data. CLDN-2 was not expressed in the f-MIX samples at 32 h time point.

There were no effects on gene expression greater than a fold change of 2.0 or lower than a fold change of 0.5 observed following f-2’FL/Tw3 and f-2’FL/C 24 h treatments (Figure 5A). For f-MIX/T and f-MIX/C 24 h treatments, a ~0.25-fold reduction in CLDN-2 gene expression was observed in treatment versus the control (Figure 5B), whereas no effect within this fold range was observed for the other target genes tested (Figure 5B). The 2^(−∆∆Ct) results were consistent for all three reference genes (Figure 5A,B). The gene expression data for f-2’FL were consistent with the ELISA data reported above for cytokines IL-8 and GRO-α, whereas IL-6 showed a reduction in protein amount and no substantial effect on gene expression (Figure 4). Gene expression data for f-MIX were not consistent with protein data for cytokines (Figure 4). Of note, gene expression of CLDN-2, -5, and -8 was generally low, with Ct vales of ≥35.

Interestingly, following the 32 h treatment, a significant increase in expression of CLDN-8 gene was observed when Caco2 monolayers were treated with f-2’FL/Tw3 (≥4.17-fold) and f-MIX/T (≥3.92-fold), with no effect on expression of other target genes (Figure 5A,B). Again, the 2^(−∆∆Ct) results were consistent for all three housekeeping genes (Figure 5A,B). CLDN-2 was not expressed at this time point.

To further assess the effectiveness of f-HMOs in strengthening gut barrier function, proximal, transverse, and distal Colon Intestine-Chips were generated from human intestinal biopsies and cultured under microfluidic conditions. Colon Intestine Chip is an advanced in vitro model of human large intestine based on microfluidic organs-on-chip technology developed by Emulate Inc. Colon Intestine Chip recreates human intestinal physiology by incorporating key primary cell types in distinct epithelial and vascular channels [48]. Organoid-derived primary epithelial cells are cultured on top of an extracellular matrix protein-coated porous membrane, and primary intestinal microvascular endothelial cells are cultured in the vascular channel. Cyclic stretch is applied to the membrane, recreating in vivo relevant mechanical forces that emulate intestinal tissue functions and architecture [49]. A tight epithelial monolayer was established in the Colon Intestine-Chips, on day 2 post seeding, as indicated by the apparent permeability of FD4 (<0.5 × 10^−6^ cm/s) [50] (Figure S8A,B). Following treatment of the gut-on-chip with f-2’FL/C and f-2’FL/Tw3 for 32 h, the expression of genes involved in promoting integrity of barrier function and immune response were determined as above.

**Figure 6:**
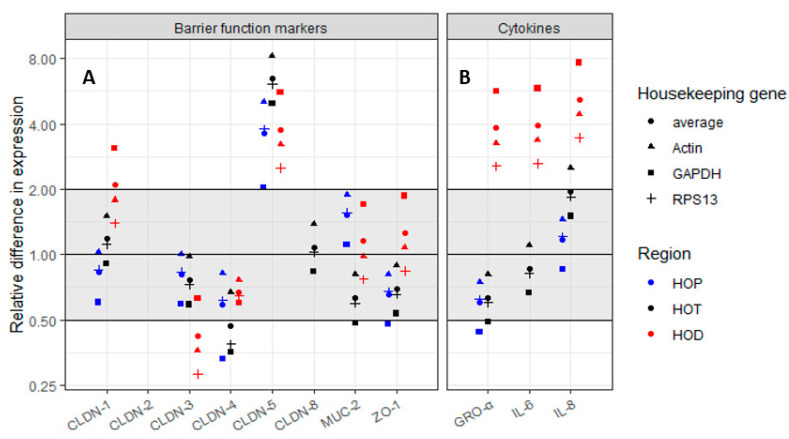
Effect of the f-HMO treatment on gene expression of barrier function proteins of the gut-on-chip. (**A**) Effect of 32 h treatment with f-2’FL on gene expression of barrier function proteins of proximal (HOP), transverse (HOT), and distal gut-on-chips (HOD); (**B**) Effect of 32 h treatment with f-2’FL on gene expression of cytokines and chemokines. Data are shown using 2^(−∆∆Ct) method. Actin, GAPDH, and RPS13 were used as reference genes to normalize the data. CLDN-2 was not expressed in any of the three gut-on-chips. CLDN-8 expression could not be determined in HOP and HOD, and IL-6 in HOP.

As shown in Figure 6**,** treatment with f-2’FL/Tw3, for 32 h, led to an upregulation of CLDN-5 expression from 2.03- to 8.25-fold across all three Colon Intestine-Chips (proximal, transverse, and distal). This was consistent across the three housekeeping genes tested (GAPDH, RPS13, and actin). Some differences were observed for other target genes such as CLDN-1, CLDN-3, or CLDN-4, but not consistently across the three housekeeping genes. It is worth noting that CLDN-2 was not expressed in any of the Colon Intestine-Chips and that CLDN-8 expression was generally very low, as shown by Ct values >35 for transverse chip. CLDN-8 expression could not be determined in proximal and distal chips.

Treatment with f-2’FL/Tw3 had no substantial effect on IL-6 and IL-8 in proximal and transverse Colon Intestine-Chips, as shown by ELISA (Figures S9 and S10) or gene expression analyses (Figure 6). IL-6 gene expression in proximal chip could not be determined, which is consistent with low IL-6 amounts assessed with ELISA. There was also no difference in GRO-α between f-2’FL/Tw3 and the control in proximal and transverse chips (Figure 6). The distal Colon Intestine Chip showed an increase in expression of ~3.93-fold for IL-6 gene, 5.18-fold for IL-8 gene, and 3.83-fold for GRO-α gene following treatment with f-2’FL/Tw3 as compared with f-2’FL/C (Figure 6 and Table S5). These gene expression results are in line with the trend in increased protein amounts of IL-6 and IL-8 (*p* = 0.084 and 0.127, respectively), as shown by ELISA (Figures S9 and S10).

## 4. Discussion

HMOs are complex carbohydrates highly abundant in human milk. To date, approximately 200 different oligosaccharide structures have been identified in breast milk. These structures can be categorized into three principal classes of HMOs; neutral core, sialylated, and fucosylated [51]. Their concentrations in maternal milk vary according to factors such as day of lactation, mother’s genetic secretor status, and birth type, but large interlaboratory variations have also been reported as reviewed previously [52]. HMOs of different structures differentially affect the function of the gut barrier of the infant helping to maintain gut health [53]. A recent clinical trial in adults showed that supplementing the diet with HMOs could be a valuable strategy to shape the human gut microbiota and specifically promote the growth of beneficial bifidobacteria [18]. Supplementation of 2’FL and LNnT at daily doses up to 20 g was shown to be safe and well tolerated, as assessed using the GI symptoms rating scale. HMO supplementation specifically modified the adult gut microbiota with the primary impact being substantial increases in relative abundance of actinobacteria and bifidobacteria in particular and a reduction in relative abundance of Firmicutes and Proteobacteria [18].

Here, the SHIME^®^ system inoculated with fecal samples from one healthy donor was run for three weeks to gain mechanistic insights into the role of 2’FL, LnNT, and MIX on the adult microbiome and gut barrier. The decision to focus on a single donor was justified by a pretest where the mechanism of HMO fermentation over five human donors was found to be consistent in terms of modulation of microbial activity and bifidogenic effects. Moreover, interindividual differences are reduced in the long-term SHIME^®^ model as the factors that drive interindividual differences (i.e., differences in baseline diet or physiological parameters (pH, transit time, enzyme levels, and bile salt levels)) are highly standardized. The long-term model, thus, served to provide mechanistic insight under more controlled conditions. Using this system, we showed that 2’FL and MIX led to an increase in the level of bifidobacteria concurring with an increase of acetate at Week 1. The most dominant bifidobacterial taxa detected in the adult human gut are *B. longum subsp. longum*, *B. pseudocatenulatum*, *B. adolescentis*, and *B. catenulatum* [54]. Although it is not yet known which bifidobacterial species from adults can utilize HMOs, strains of *Bifidobacterium longum* and *B. pseudocatenulatum* isolated from infants have been shown to metabolize 2’FL [55,56,57]. The impact of 2’FL fermentation resulted in cross feeding interaction with bifidobacteria as initial degraders and butyrate producers such as *B. coccoides*/*E. rectale* as secondary degraders. This cross feeding was accompanied by a change in the metabolite profile during the three weeks of treatment with an increase in butyrate production at Week 3. Such an example of HMO cross feeding was reported in in vitro co-cultures, where *E. hallii* utilized acetate and lactate from fermentation of 2’FL by *Bifidobacterium infantis* leading to production of butyrate [58].

To date, only a limited number of studies have investigated the effects of HMOs on intestinal barrier function. Observations in rodents showed that GOS or probiotic Bifidobacterium strains could improve intestinal permeability and restore mucus function [59,60] and a recently-published animal study showed that 2’FL- and 3’SL-fortified diets altered gut microbiota composition and gene expression in the GI tract of rats [61]. However, due to intrinsic differences in the GI physiology, mucin glycosylation and microbiota of mice and humans [62], observations in rodents might not always translate to humans. Here, we investigated the mechanisms of 2’FL and LNnT, and combination thereof, on gut barrier function. First, we used Caco2 cells to perform an initial permeability assay using fermented HMOs. We showed that f-2’FL and f-MIX treatments led to a reduction in FD4 permeability using Caco2 cell monolayers grown on transwells, suggesting a strengthening of the gut barrier. Several proteins contribute to the development of tight junctions, i.e., integral membrane proteins which include claudins, junctional complex proteins (zonula occludens and other cytoplasmic proteins), as well as cell cytoskeleton structures. Claudin-1, -3, -4, -5, and -8 strengthen the barrier, whereas claudin-2, -7, and-12 weaken it [19]. ZO-1 is also important for barrier function [26,63]. We showed a significant increase in expression of the CLDN-8 gene (3.92–6.13-fold) when differentiated Caco2 monolayers were treated for 32 h with f-2’FL/Tw3 and f-MIX. The impact of the treatment was time dependent as no effect on CLDN-8 expression was observed after 24 h treatment, whereas a reduction in CLDN-2 expression was observed after 24 h MIX/T treatment but not at 32 h. These changes in claudin expression are in favor of a strengthening of the barrier. Furthermore, IL-6 was the main cytokine showing a consistent reduction in secretion for all f-HMOs tested on Caco2 monolayers, whereas the impact on IL-8, GRO-α, or TGF-β was f-HMO specific, as determined by multiplex ELISA. These data are in line with a previous study showing that IL-6 enhances expression of CLDN-2 using Caco2 cells, resulting in an increase in tight junction permeability [64]. It is possible that these observations reveal novel mechanisms by which IL-6 can underpin the integrity of the intestinal barrier by modulating claudin expression. However, more work is required to confirm the changes of expression of tight junction proteins upon f-HMO treatment at the protein level.

One of the limitations of Caco2 monolayer models is that they do not recapitulate the properties of the intestinal mucus layers in the intestine (as expressing different mucins, displaying cancer-type glycosylation, and not forming a mucus gel), and therefore are not representative of the barrier integrity in vivo. To further assess the impact of fermented HMOs on the gut barrier function, gut-on-chips were generated using human organoids derived from proximal, transverse, and distal colon biopsies. The gut-on-chip is a microengineered platform that can be used for the co-culture of intestinal epithelial cells derived from organoids and intestinal tissue-specific microvascular endothelial cells under microfluidic conditions [48,49]. Using this advanced model, we showed that CLDN-5 was significantly upregulated across proximal, transverse, and distal Colon Intestine-Chips (from 2.03- to 8.25-fold) following treatment with fermented 2’FL at Week 3, for 32 h. Expression of CLDN-5 was low in the proximal colon and increased from transverse to distal. The differences in basal expression of the tight-junction proteins between the models (such as the low expression of CLDN-8 in Colon Intestine-Chips) could account for the differences observed for fermented 2’FL between Caco2 and gut-on-chip models. Another parameter to consider is the influence of the mechanical forces applied to the gut-on-chip system as compared with the Caco2 cells under static culture. A recent study highlighted that applying shear force equivalent to that experienced during intestinal peristalsis to Caco2 cells increased the modulating effects of HMOs on the gene expression of tight junction proteins [65], further supporting the importance of mechanical forces, such as flow and stretch to emulate the dynamic cellular microenvironment.

The impact of fermented 2’FL on gut permeability is associated with a change in metabolite profiles in the SHIME^®^ system showing an increase in butyrate production at Week 3. The decrease in permeability for fermented 2’FL at Week 3 was similar to that of butyrate used as a control. These results are consistent with previous independent studies using Caco2 cells showing (i) that butyrate could significantly increase the transepithelial electrical resistance [66] and induce expression of tight junctions [67] and (ii) that stimulating the growth of bifidobacteria by HMOs but not lactose could enhance tight junction protein expression [68]. Here, we showed that 2’FL cross feeding in the adult gut microbiota results in the strengthening of the gut barrier in Caco2 cells and in the Colon Intestine-Chips. This effect could be explained, at least in part, by the increased level of butyrate in these samples. The decrease in permeability observed for the fermented 2’FL and MIX, but not fermented LNnT, could be due to the contribution of bifidobacteria which was substantially increased following MIX and 2’FL treatments.

Collectively, our data using highly standardized models suggest that fermented HMOs by adult gut microbiota can beneficially modulate gut barrier function but with differential impact on permeability, cytokines, and tight junction proteins. From the range of fermentation conditions tested in this work, fermented 2’FL at Week 3 showed the most beneficial impact, with a reduced permeability on Caco2 monolayers and upregulation of tight junction proteins, claudin-8 and claudin-5, on Caco2 cells and human-based Colon Intestine-Chips, respectively, supporting a strengthening of gut barrier function in adults. We showed the impact of f-HMOs where interindividual differences were reduced; independent replication with multiple donors would strengthen the observed treatment effects. Together, with the demonstrated safety, tolerance, and impact of HMOs on the adult gut microbiota in clinical trials, this study supports the potential use of HMOs as a novel strategy to restore or promote gut barrier function in adults.

## Supplementary Materials

The following are available online at https://www.mdpi.com/2072-6643/12/9/2808/s1, Figure S1: HMO structures of 2’-fucosyllactose (2’FL) and lacto-N-neotetraose (LNnT), Figure S2: Schematic of SHIME^®^ experimental design, Figure S3: Analysis of RNA quality, Figure S4: Prescreening of HMO prebiotic effect, Figure S5: FD4 permeability of differentiated Caco2 monolayers treated with f-HMOs, Figure S6: Apical cytokine amounts secreted by Caco2 cells following f-HMO treatment, Figure S7: Basolateral cytokine amounts secreted by Caco2 cells following f-HMO treatment, Figure S8: Characterization of human colonic monolayer on Colon Intestine Chip, Figure S9: IL-6 cytokine secretion of Colon Intestine-Chips following treatment with f-HMOs, Figure S10: IL-8 cytokine secretion of Colon Intestine-Chips following treatment with f-HMOs, Table S1: List of primers used in this study, Table S2: Clinical details of biopsy donors, Table S3: Impact of f-HMOs on FD4 permeability in Caco2 cells, Table S4: Impact of f-HMOs on cytokine production in Caco2 cells, Table S5: Gene expression analysis of barrier function markers and cytokines using Colon Intestine-Chips treated with f-2’FL/w3 and f-2’FL/C.
