# Supplementary material for: Effects of Human Milk Oligosaccharides on the Adult Gut Microbiota and Barrier Function
Source: Nutrients. 2020 Sep 13;12(9):2808. doi: 10.3390/nu12092808 (PMC7551690; doi:10.3390/nu12092808)
Supplement: Supplementary file 1 [file nutrients-12-02808-s001.pdf]

Supplementary Figures

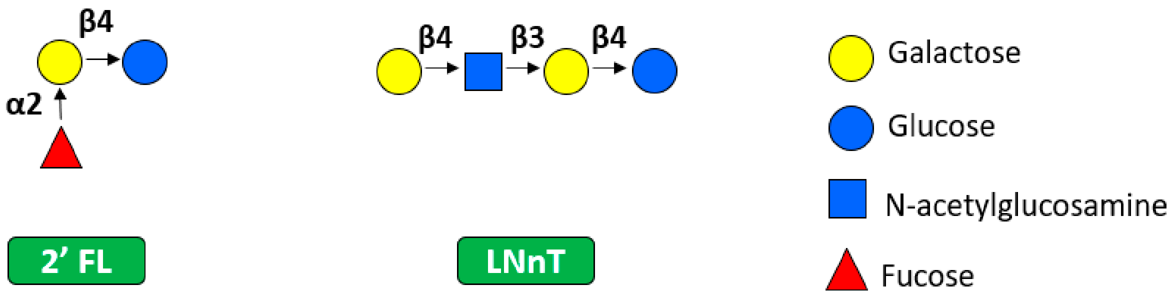

Figure S1: HMO structures of 2'-Fucosyllactose (2’FL) and Lacto-N-neotetraose (LNnT). The lactose core is fucosylated (2’FL) or linked to N-acetyllactosamine (GalNAc) and galactose (LNnT).

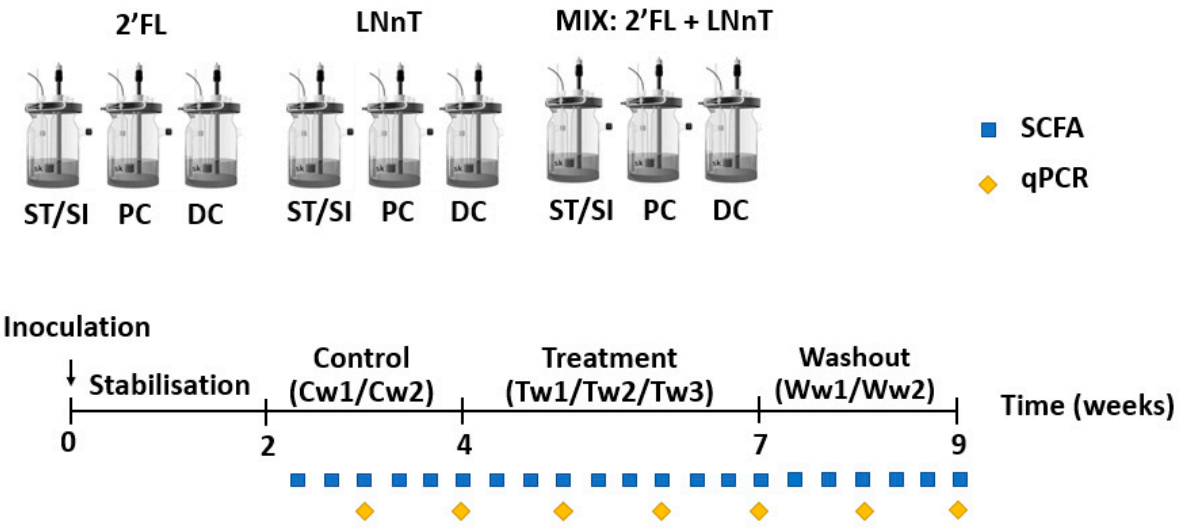

Figure S2: Schematic of SHIME experimental design. Treatment with HMOs included 2’FL, LNnT and the mix of 2’FL and LNnT (MIX). ST/SI stands for stomach/small intestine vessel, PC proximal colon vessel and DC distal colon vessel. Timeline of sampling for SCFA and microbial population analyses is shown over control period for week 1 and 2 (Cw1 and Cw2), treatment period for weeks 1 to 3 (Tw1, Tw2 and Tw3) and washout period for 2 weeks (Ww1 and Ww2). SCFA analysed at these points include acetate, propionate and butyrate. Quantitative PCR was performed to enumerate Bifidobacteria, *B. coccoides/E. rectale*, Firmicutes and Bacteroidetes.

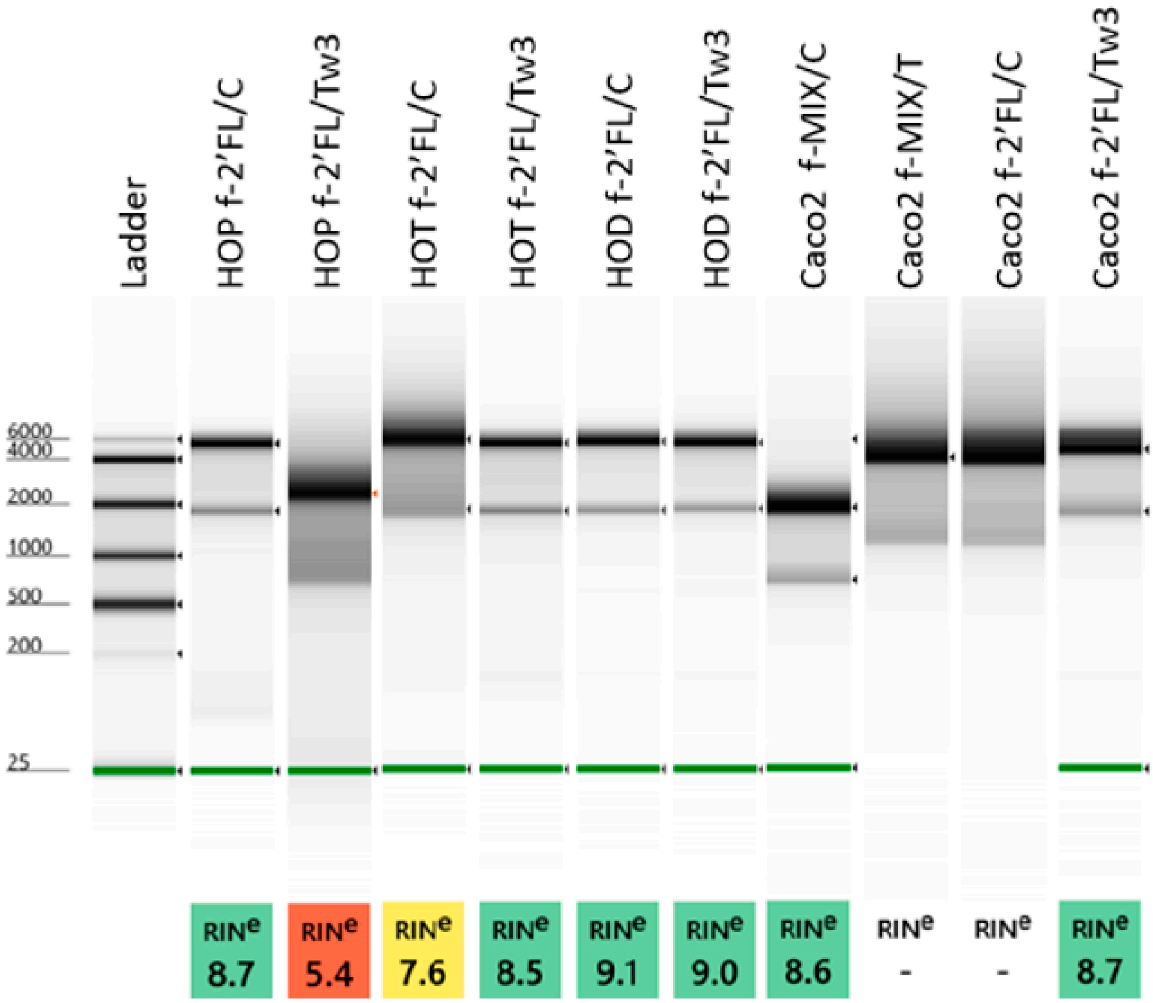

Figure S3: Analysis of RNA quality. RNA was extracted from gut-on-chips HOP, HOT and HOD or from Caco2 cells treated with f-2'FL/C and f-2'FL/Tw3 and from Caco2 cells treated with f-MIX/C and f-MIX/T. All samples were treated with f-HMOs for 32 h and 12-137 ng/µL RNA was analysed using the Tapestation.

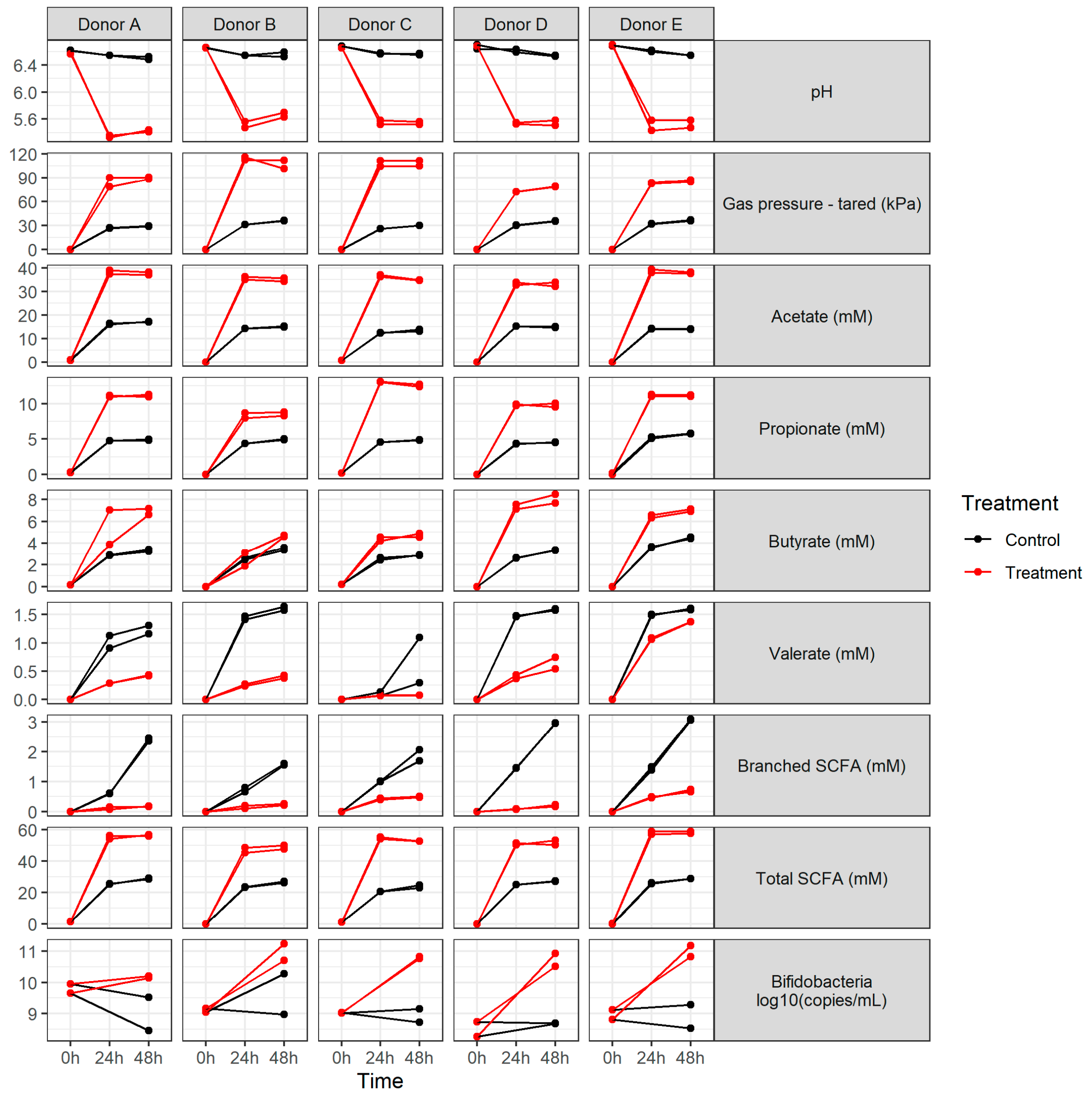

Figure S4: Pre-screening of HMO prebiotic effect. Short-term colonic simulations (48 h) with 2'FL/LNnT (4:1 ratio) at 5g/L were carried out with faecal samples from 5 donors to evaluate potential inter-individual differences to 2FL/LNnT in terms of pH decrease, gas production, short chain fatty acid production and bifidogenic effects. Negative control contained only colon medium. Samples were collected at the beginning, and on 24 h and 48 h after the start of the incubation. Both the control incubations and the treatments were performed in duplicate per donor.

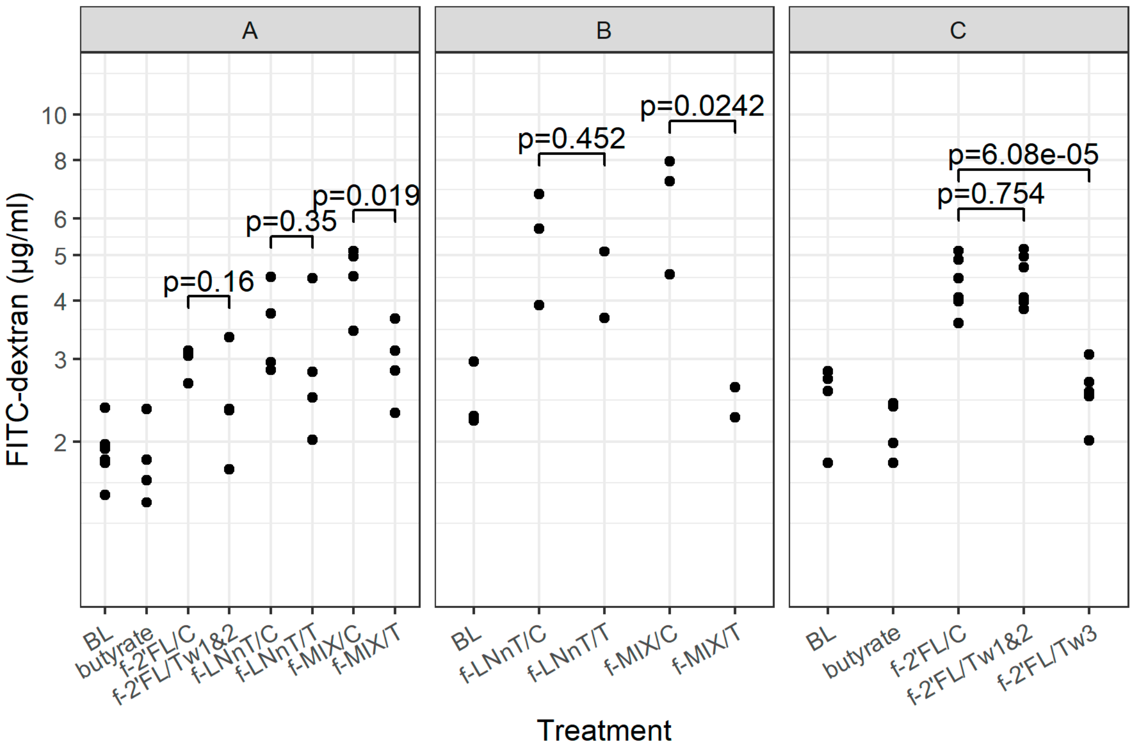

Figure S5: FD4 permeability of differentiated Caco2 monolayers treated with f-HMOs. Data showed results from 3 individual experiments (panels A, B and C). Pairwise t-tests using log-transformed data were conducted, p-values are shown.

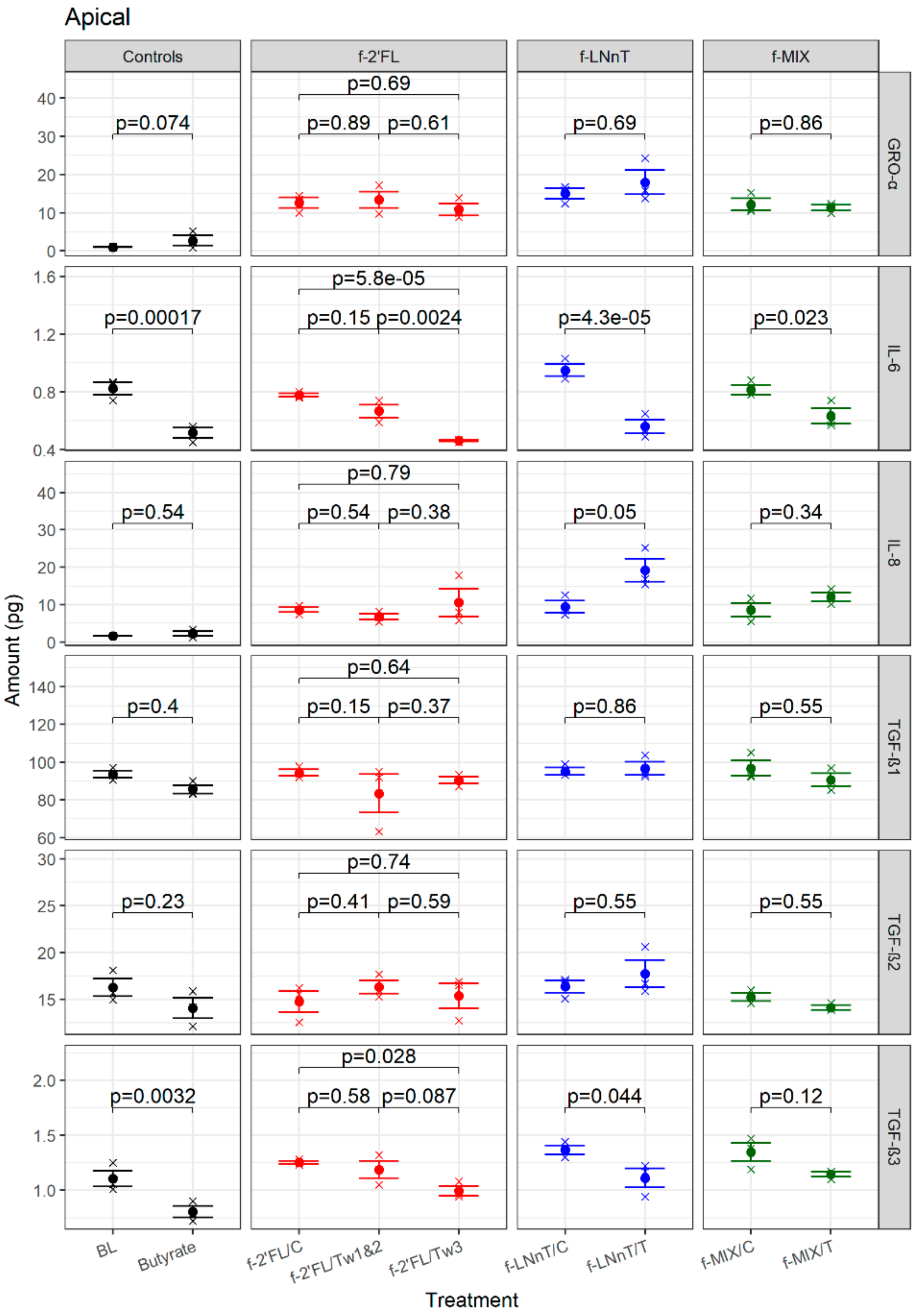

Figure S6: Apical cytokine amounts secreted by Caco2 cells following f-HMO treatment. Cytokines analysed included GRO-α, IL-6, IL-8 and TGF-β1-3 following treatment with f-2'FL, f-LNnT and f-MIX for 24 h. Medium only treated cells (BL) and 5 mM butyrate treated cells were included as controls, p values are shown.

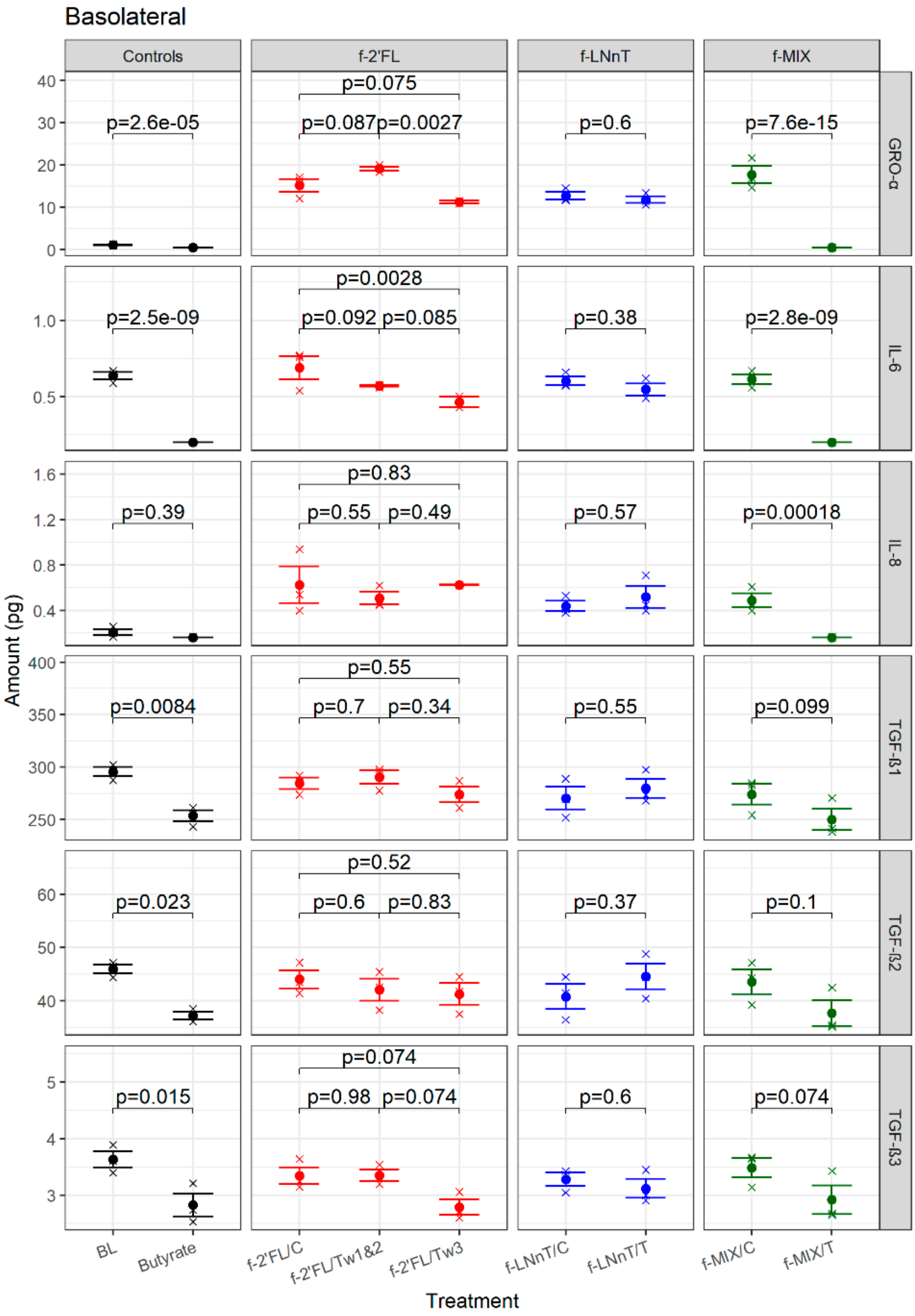

Figure S7: Basolateral cytokine amounts secreted by Caco2 cells following f-HMO treatment. Cytokines analysed included GRO-α, IL-6, IL-8 and TGF-β1-3 following treatment with f-2'FL, f-LNnT and f-MIX for 24 h. Medium only treated cells (BL) and 5 mM butyrate treated cells were included as controls, p values are shown.

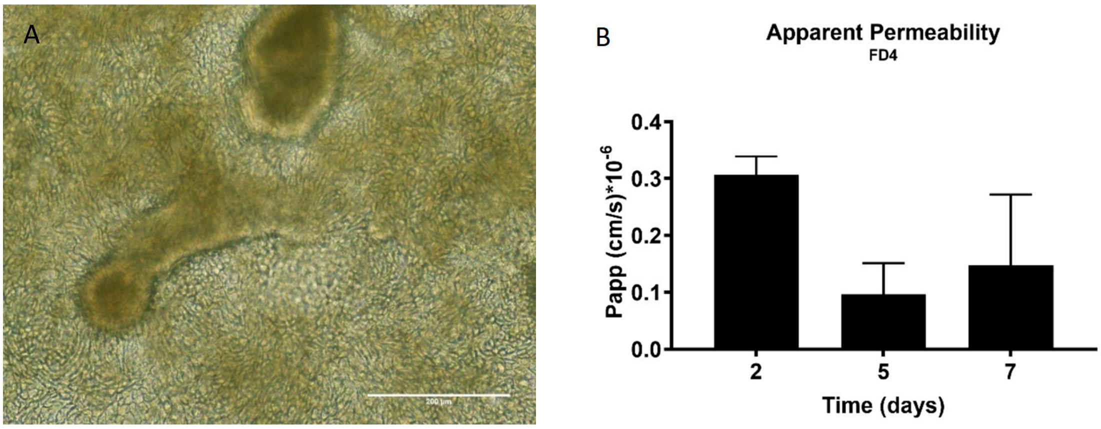

Figure S8: Characterisation of human colonic monolayer on Colon Intestine-Chip. A) Primary human colonic monolayer shows differentiated plaque-like structure on day 7 including 3D structures, bar size 200 µm. B) Apparent permeability of the monolayer assessed on day 2, 5 and 7 post-seeding the chips.

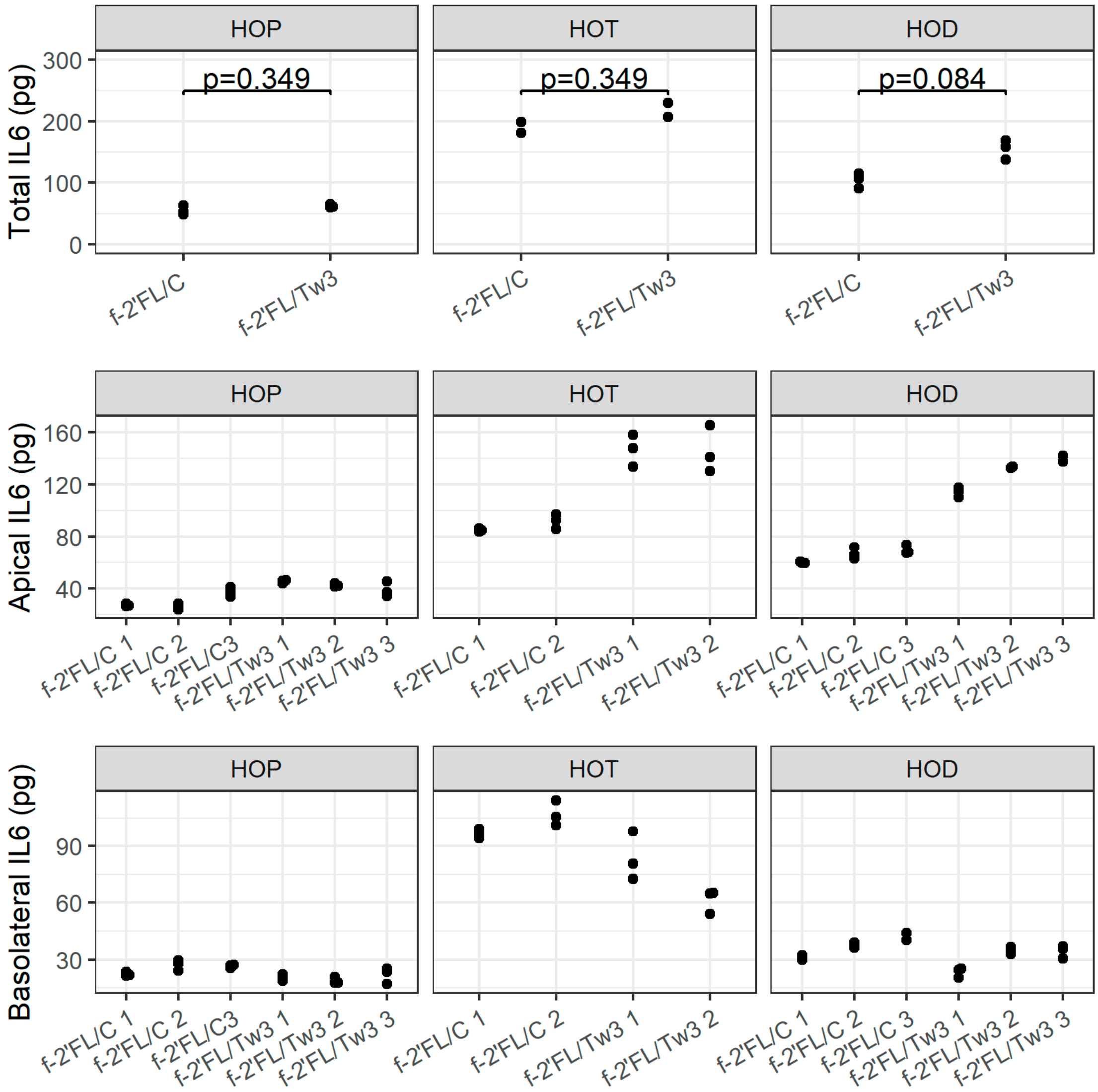

Figure S9: IL-6 cytokine secretion of Colon Intestine-Chips following treatment with f-HMOs. A) total IL-6 amounts, B) apical IL-6 and C)/ basolateral IL-6 secreted after 24 h treatment with f-2’FL/C and f-2’FL/Tw3 of proximal (HOP), transverse (HOT) and distal (HOD) Colon Intestine-Chips. Experiments were performed in triplicates for HOP and HOD chips and duplicates for HOT chips, p values are shown for total cytokine secretion.

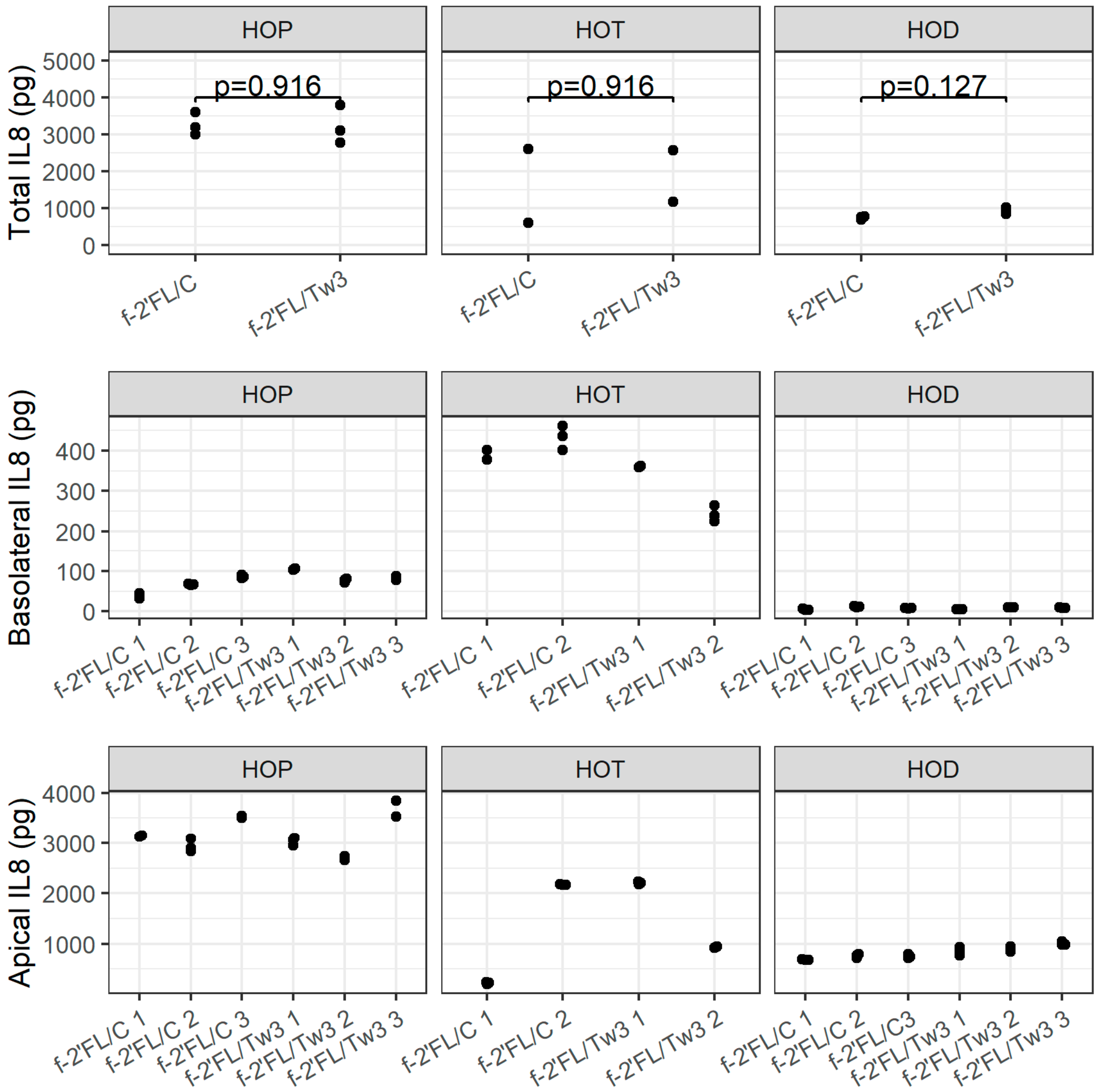

Figure S10: IL-8 cytokine secretion of Colon Intestine-Chips following treatment with f-HMOs. Top panel shows total IL-8 amounts, middle panel apical IL-8 and bottom panel basolateral IL-8 secreted after 24 h treatment with f-2'FL/C and f-2'FL/Tw3 of proximal (HOP), transverse (HOT) and distal (HOD) Colon Intestine-Chips. Experiments were performed in triplicates for HOP and HOD chips and duplicates for HOT chips, p values are shown for total cytokine secretion.

Supplementary Tables

Table S1: List of primers used in this study

Target	Primer	Catalogue number / Sequence (5'-3')
Bifidobacterium spp.	F	TCGCGTC(C/T)GGTGTGAAAG
	R	CCACATCCAGC(A/G)TCCAC
Clostridium coccoides–	F	CGGTACCTGACTAAGAAGC
Eubacteriumrectale group	R	AGTTT(C/T)ATTCTTGCGAACG
Bacteroidetes	F	GGARCATGTGGTTTAATTCGATGAT
	R	AGCTGACGACAACCATGCAG
Firmicutes	F	GGAGYATGTGGTTTAATTCGAAGCA
	R	AGCTGACGACAACCATGCAC
IL-6	Hs_IL6_1_SG QT Primer Assay	QT00083720
CLDN-2	Hs_CLDN2_1_SG QT Primer Assay	QT00089481
CLDN-3	Hs_CLDN3_1_SG QT Primer Assay	QT00201376
CLDN-4	Hs_CLDN4_1_SG QT Primer Assay	QT00241073
CLDN-5	Hs_CLDN5_1_SG QT Primer Assay	QT00232197
CLDN-8	Hs_CLDN8_1_SG QT Primer Assay	QT00212268
CLDN-1	F	GCAGATCCAGTGCAAAGTCT
	R	CATACACTTCATGCCAACGG
ZO-1	F	AAGTCACACTGGTGAAATCC
	R	CTCTTGCTGCCAAACTATCT
Actin	F	TGACGTGGACATCCGCAAAG
	R	CTGGAAGGTGGACAGCGAGG
GAPDH	F	AGGTCGGAGTCAACGGATTT
	R	TGGAAGATGGTGATGGGATTT
GRO-α	F	GCCAGTGCTTGCAGACCCT
	R	GGCTATGACTTCGGTTTGGG
IL-8	F	TTGAGAGTGGACCACACTGC
	R	TGCACCCAGTTTTCCTTGG
RPS13	F	CGAAAGCATCTTGAGAGGAACA
	R	TCGAGCCAAACGGTGAATC
MUC-2	F	ACTGCACATTCTTCAGCTGC
	R	ATTCATGAGGACGGTCTTGG

Table S2: Clinical details of colon biopsy donors

Age (y)	Sex	Reason for referral	Clinical history and diagnosis	Colon part for biopsy	Sample code*
38	M	Cancer surveillance follow up	Cancer on a rectal polyp in 2015. Endoscopies in 2016 and 2019 were normal.	Proximal colon	HOP
60	M	Family history of colorectal cancer	Two hyperplastic polyps in proximal colon. No evidence of dysplasia or malignancy.	Transverse colon	HOT
63	M	Family history of colorectal cancer	One splenic flexure and one transverse tubular adenoma polyp. Low grade dysplasia, no malignancy.	Distal colon	HOD

*Biopsies from macroscopically normal regions of proximal, transverse and distal colon (HOP, HOT and HOD) were donated by 3 study participants diagnosed with polyps.

Table S3: Impact of f-HMOs on FD4 permeability in Caco2 cells

Sample	Period	Ratio compared to BL		Ratio treated vs control*	
		Ratio (95% CI[a])	p-value	Ratio (95% CI)	p-value
**f-2'FL**	C	1.63 (1.37 - 1.93)	9.31e-07	Ref.[b]	
	Tw1 & Tw2	1.51 (1.27 - 1.80)	1.82e-05	0.91 (1.09 - 0.76)	0.306
	Tw3	0.98 (0.79 - 1.21)	0.846	0.59 (0.73 - 0.48)	1.54e-05
**f-LNnT**	C	2.00 (1.65 - 2.43)	3.73e-09	Ref.[b]	
	T	1.60 (1.31 - 1.96)	2.55e-05	0.80 (1.00 - 0.64)	0.0703
**f-MIX**	C	2.50 (2.06 - 3.04)	6.14e-13	Ref.[b]	
	T	1.36 (1.11 - 1.67)	0.0046	0.56 (0.70 - 0.45)	8.92e-06

*Ratio compared to medium only (BL) is shown and each treatment samples versus its control sample.

[a]CI confidence intervals.

[b]Ref stands for values which are non-applicable (ratios of control HMO versus its control and associated p-values).

Table S4: Impact of f-HMOs on cytokine production in Caco2 cells.

Control	Treatment	IL6		TGFb1		TGFb2		TGFb3		IL8		GROa	
**Total**		**Ratio (95% CI)**	**p-value**	**Ratio (95% CI)**	**p-value**	**Ratio (95% CI)**	**p-value**	**Ratio (95% CI)**	**p-value**	**Ratio (95% CI)**	**p-value**	**Ratio (95% CI)**	**p-value**
BL	Butyrate	0.49 (0.44-0.55)	1.79E-08	0.87 (0.82-0.93)	0.00267	0.82 (0.75-0.90)	0.00285	0.76 (0.67-0.88)	0.00612	1.23 (0.77-1.96)	0.552	1.27 (0.81-1.99)	0.492
2'FL/C	2'FL/Tw1&2	*0.84 (0.75-0.94)	0.0329	0.99 (0.93-1.05)	0.759	0.99 (0.91-1.08)	0.859	0.98 (0.86-1.13)	0.859	0.78 (0.49-1.25)	0.494	1.17 (0.74-1.82)	0.607
2'FL/C	2'FL/Tw3	0.63 (0.56-0.72)	2.97E-05	0.96 (0.90-1.02)	0.388	0.96 (0.88-1.05)	0.552	0.82 (0.72-0.95)	0.0446	1.38 (0.82-2.32)	0.404	0.81 (0.49-1.34)	0.553
2'FL/Tw1&2	2'FL/Tw3	0.75 (0.66-0.85)	0.00274	0.97 (0.92-1.04)	0.552	0.97 (0.89-1.06)	0.63	0.84 (0.73-0.96)	0.0659	1.76 (1.05-2.96)	0.11	0.70 (0.42-1.15)	0.344
LNnT/C	LNnT/T	0.71 (0.63-0.80)	0.000169	1.03 (0.97-1.10)	0.538	1.09 (1.00-1.19)	0.152	0.91 (0.79-1.04)	0.35	2.00 (1.26-3.18)	0.0368	1.06 (0.68-1.66)	0.848
MIX/C	MIX/T	0.58 (0.52-0.65)	8.47E-07	0.92 (0.86-0.98)	0.0476	0.88 (0.80-0.96)	0.0434	0.84 (0.73-0.96)	0.0676	1.39 (0.87-2.21)	0.35	0.40 (0.25-0.62)	0.00471
**Apical**													
BL	Butyrate	0.63 (0.54-0.73)	0.000169	0.91 (0.79-1.05)	0.397	0.86 (0.73-1.02)	0.229	0.73 (0.62-0.84)	0.00323	1.28 (0.76-2.16)	0.538	2.08 (1.16-3.76)	0.0736
2'FL/C	2'FL/Tw1&2	0.85 (0.73-1.00)	0.148	0.87 (0.75-1.00)	0.15	1.11 (0.93-1.32)	0.411	0.94 (0.81-1.10)	0.575	0.78 (0.46-1.31)	0.538	1.05 (0.58-1.89)	0.89
2'FL/C	2'FL/Tw3	0.60 (0.51-0.70)	5.75E-05	0.96 (0.83-1.10)	0.641	1.04 (0.88-1.23)	0.74	0.79 (0.68-0.92)	0.0284	1.10 (0.65-1.85)	0.792	0.85 (0.47-1.54)	0.688
2'FL/Tw1&2	2'FL/Tw3	0.70 (0.60-0.82)	0.00244	1.10 (0.95-1.27)	0.37	0.94 (0.79-1.11)	0.586	0.84 (0.72-0.98)	0.0872	1.41 (0.84-2.37)	0.384	0.82 (0.45-1.47)	0.606
LNnT/C	LNnT/T	0.59 (0.50-0.69)	4.25E-05	1.02 (0.88-1.17)	0.859	1.08 (0.91-1.28)	0.552	0.81 (0.69-0.94)	0.0442	2.04 (1.21-3.44)	0.0499	1.17 (0.65-2.11)	0.688
MIX/C	MIX/T	0.77 (0.66-0.91)	0.0234	0.94 (0.81-1.08)	0.552	0.93 (0.78-1.10)	0.552	0.85 (0.74-0.99)	0.124	1.46 (0.86-2.45)	0.344	0.94 (0.52-1.69)	0.859
**Basolateral**													
BL	Butyrate	0.31 (0.27-0.37)	2.49E-09	0.86 (0.79-0.93)	0.00837	0.81 (0.71-0.92)	0.0234	0.78 (0.67-0.90)	0.0153	0.79 (0.55-1.14)	0.388	0.47 (0.38-0.57)	2.62E-05
2'FL/C	2'FL/Tw1&2	0.84 (0.72-0.98)	0.0925	1.02 (0.94-1.11)	0.696	0.95 (0.84-1.09)	0.604	1.00 (0.86-1.16)	0.978	0.86 (0.60-1.23)	0.552	1.28 (1.04-1.57)	0.0872
2'FL/C	2'FL/Tw3	0.68 (0.57-0.81)	0.00276	0.96 (0.89-1.04)	0.548	0.94 (0.82-1.07)	0.522	0.83 (0.72-0.97)	0.0739	1.06 (0.71-1.59)	0.828	0.75 (0.60-0.95)	0.0754
2'FL/Tw1&2	2'FL/Tw3	0.81 (0.68-0.97)	0.0846	0.94 (0.87-1.02)	0.344	0.98 (0.86-1.12)	0.828	0.83 (0.72-0.96)	0.0736	1.24 (0.83-1.86)	0.494	0.59 (0.47-0.74)	0.00267
LNnT/C	LNnT/T	0.90 (0.77-1.06)	0.385	1.04 (0.95-1.12)	0.552	1.09 (0.96-1.25)	0.37	0.95 (0.82-1.10)	0.604	1.16 (0.81-1.66)	0.567	0.93 (0.75-1.14)	0.604
MIX/C	MIX/T	0.33 (0.28-0.38)	2.84E-09	0.91 (0.84-0.99)	0.0994	0.86 (0.76-0.99)	0.1	0.83 (0.72-0.97)	0.0739	0.34 (0.24-0.49)	0.000176	0.03 (0.02-0.04)	7.56E-15

*Ratios of cytokine amounts for treatment vs control.

Table S5: Gene expression analysis of barrier function markers and cytokines using Colon Intestine-Chips treated with f-2'FL/w3 and f-2'FL/C

A) Barrier function markers

	CLDN-1			CLDN-2			CLDN-3			CLDN-4			CLDN-5			CLDN-8			ZO-1			MUC-2		
	[a]HOP	HOT	HOD	HOP	HOT	HOD	HOP	HOT	HOD	HOP	HOT	HOD	HOP	HOT	HOD	HOP	HOT	HOD	HOP	HOT	HOD	HOP	HOT	HOD
Average[b]	0.83	1.18	2.09	[c]ud	ud	ud	0.81	0.76	0.42	0.59	0.47	0.67	3.62	6.45	3.76	ud	1.08	ud	0.65	0.69	1.26	1.52	0.63	1.15
GAPDH	0.60	0.91	3.09	ud	ud	ud	0.59	0.59	0.62	0.33	0.35	0.60	2.03	4.97	5.56	ud	0.83	ud	0.48	0.53	1.86	1.11	0.49	1.70
RPS13	0.85	1.12	1.40	ud	ud	ud	0.83	0.72	0.28	0.61	0.39	0.65	3.78	6.12	2.51	ud	1.02	ud	0.67	0.66	0.84	1.56	0.60	0.77
Actin	1.03	1.51	1.78	ud	ud	ud	1.00	0.98	0.36	0.82	0.67	0.76	5.07	8.25	3.21	ud	1.38	ud	0.81	0.89	1.07	1.88	0.81	0.98

B) Cytokines

	IL-6			IL-8			GRO-α		
	[a]HOP	HOT	HOD	HOP	HOT	HOD	HOP	HOT	HOD
Average[b]	[c]ud	0.86	3.93	1.17	1.95	5.18	0.60	0.63	3.83
GAPDH	ud	0.66	5.82	0.86	1.50	7.66	0.44	0.49	5.66
RPS13	ud	0.82	2.62	1.21	1.85	3.46	0.62	0.60	2.55
Actin	ud	1.10	3.36	1.46	2.49	4.42	0.75	0.81	3.26

[a]HOP, proximal HOT, transverse and HOD distal Colon Intestine-Chips

[b]Geometric mean of the qPCR results is shown as average using the $2^{-(\Delta\Delta Ct)}$ method. Data were normalised to GAPDH, RPS13 and actin reference genes.

[c]Ud means the Ct values were undetermined.
